# Magnetic fields in non-convective regions of stars

**DOI:** 10.1098/rsos.160271

**Published:** 2017-02-22

**Authors:** Jonathan Braithwaite, Henk C. Spruit

**Affiliations:** 1 Argelander Institut für Astronomie Universität Bonn, Auf dem Hügel 71, 53121 Bonn; 2 Max Planck Institut für Astrophysik Karl-Schwarzschild-Str. 1, 85741 Garching

**Keywords:** stars: magnetic fields, stars: neutron, magnetohydrodynamics

## Abstract

We review the current state of knowledge of magnetic fields inside stars, concentrating on recent developments concerning magnetic fields in stably stratified (zones of) stars, leaving out convective dynamo theories and observations of convective envelopes. We include the observational properties of A, B and O-type main-sequence stars, which have radiative envelopes, and the fossil field model which is normally invoked to explain the strong fields sometimes seen in these stars. Observations seem to show that Ap-type stable fields are excluded in stars with convective envelopes. Most stars contain both radiative and convective zones, and there are potentially important effects arising from the interaction of magnetic fields at the boundaries between them; the solar cycle being one of the better known examples. Related to this, we discuss whether the Sun could harbour a magnetic field in its core. Recent developments regarding the various convective and radiative layers near the surfaces of early-type stars and their observational effects are examined. We look at possible dynamo mechanisms that run on differential rotation rather than convection. Finally, we turn to neutron stars with a discussion of the possible origins for their magnetic fields.

## 1. Introduction

Interest in magnetic fields in the interiors of stars, in spite of a lack of immediate observability, is rapidly increasing. It is sparked by progress in spectropolarimetric observations of surface magnetic fields as well as by asteroseismology and numerical magnetohydrodynamic simulations.

An important incentive also comes from developments in stellar evolution theory. Discrepancies between results and steadily improving observations have led to a newly perceived need for evolution models ‘with magnetic fields’. At the same time, the demand for results from stellar evolution have increased for application outside stellar physics itself. An example is the need for predictable colours of stellar populations in calculations of galaxy evolution.

Key questions concern the rate of mixing of the products of nuclear burning, because stellar evolution is sensitive to the distribution of these products inside the star. The heavier nuclei are normally produced later and deeper inside the star. Outside of convective zones, these nuclei reside stably in the gravitational potential. Even weak mixing mechanisms in radiative zones, operating on long evolutionary timescales, can nevertheless change the distribution enough to affect critical stages in the evolution of stars. Possible mechanisms include hydrodynamic processes like extension of convective regions into nominally stable zones (‘overshooting’), shear instabilities due to differential rotation and large-scale circulations. Assumptions about the effectiveness of such processes are made and tuned to minimize discrepancies between computed evolution tracks and observations. The presence of magnetic fields adds new mechanisms, some of which could compete with or suppress purely hydrodynamic processes.

As well as mixing chemical elements^1^, magnetohydrodynamic processes in radiative zones should damp the differential rotation that is produced by the evolution of a star. An indirect observational clue has been the rotation rate of the end products of stellar evolution (§5.5). As the core of an evolving star contracts it tends to spin up. However, it is evident from the slow rotation of stellar remnants that angular momentum is transferred outwards to the envelope, and in many cases stellar remnants rotate much slower than can be explained even with the known hydrodynamic processes. Maxwell stresses are more effective in transporting angular momentum than hydrodynamic processes: they can even transport angular momentum across a vacuum. This leads to the study of dynamo processes driven by differential rotation in stably stratified environments (§5.4).

Note that there is a difference regarding the mixing of chemical elements. In purely hydrodynamic processes, the transport of angular momentum and chemical elements are directly related to each other, but this is not the case for magnetohydrodynamic processes. For a given rate of angular momentum transport, mixing by magnetohydrodynamic processes is less effective than in the case of hydrodynamic processes.

Until recently, the Sun was the only star for which direct measurements of *internal* rotation were available, made possible by helioseismology. For all other stars, the only source of information on angular momentum transport inside stars was the rotation of their end products. This has changed dramatically with the asteroseismic detection of rotation-sensitive oscillation modes in giants and sub-giant stars by the Kepler and CoRoT satellites. These data now provide stringent tests for theories of angular momentum transport in stars (§§5.5, 5.4).

Possible internal magnetic fields come in two distinct kinds. One kind is time-dependent magnetic fields created and maintained by some kind of dynamo process, running from some source of free energy. Dynamos in convective zones have been studied and reviewed extensively before; they are not covered in this review except for a discussion of subsurface convection in O stars (§6.5). Another obvious source of free energy is differential rotation, and this could produce a self-sustained small-scale magnetic field in a radiative zone. This would be the candidate for transport of angular momentum and chemical elements described above.

The other kind of internal magnetic field is *fossil* fields, remnants of the star formation process that have somehow survived in a stable configuration. The theory of such fields is discussed in §3.

A subset of intermediate-mass stars displays strong magnetic fields, the chemically peculiar Ap and Bp stars; and some more massive stars display similar fields. These are thought to be such fossil fields. They used to be interpreted in terms of configurations resembling simple dipoles. With the improved observations of the past decade a much larger range of configurations is found; this is reviewed in §2. Theory indicates that only a small fraction of all imaginable magnetic equilibria in a star can be stable as observed. Comparing these theoretically allowed configurations with the surface fields actually observed in individual stars gives clues about the internal structure of the fields. Together with statistical information on observed field strengths and configurations, this holds the promise of telling us something about the conditions under which the magnetic fields formed.

Though stably stratified throughout most of their interior, Ap stars still contain a small convective core. This raises the question to what extent convection interacts with the fossil field, or whether a fossil field is compatible at all with the presence of a convective zone somewhere in the star. A related question is whether stars with convective envelopes like the Sun might have fossil fields hidden in their stably stratified interiors. These questions are addressed in §6.

Also thought to be of fossil nature are the magnetic fields in neutron stars. As in upper-main-sequence stars, there is a puzzlingly enormous range in field strengths spanning five orders of magnitude. There are two obvious ways to explain this range: either it is inherited from the progenitor stars, in which case one still needs to explain the range in birth magnetic properties of main-sequence stars, or it is produced during the birth of the neutron star. It is possible that it is produced from the conversion of energy from differential rotation into magnetic, and that the same physics is at work during the birth of main-sequence stars. These issues are addressed in §7.1.

This review is organized as follows. In the next section, we look at observations of magnetic stars, with some focus on peculiarities that may hold clues on the origin of their fields. Among the main-sequence star, these are the Ap/Bp stars and the apparently non-magnetic intermediate-mass stars; next are the massive stars, and the magnetic white dwarfs then are discussed very briefly. Section 3 is a review of the theory of static ‘fossil’ magnetic fields in radiative zones, and in §4 we examine various scenarios which could explain where these fossil fields come from. In §§5 and 6, respectively, we look at the interaction of magnetic fields with differential rotation and with convection. In §7, we move onto neutron stars: their observational properties as well as likely theoretical explanations in terms of internal magnetic field. Finally, we summarize in §8.

This review goes into some depth in the magnetohydrodynamics of stars. The interested reader may wish to look at some literature on MHD, including the astrophysical context. The classic book by Roberts [1] covers basic MHD in general contexts but is out of print. More recent is the monograph by Spruit [2], tailored specifically to astrophysicists and with an emphasis on physical intuition and visualization rather than mathematics. The books by Goedbloed & Poedts [3] and Goedbloed *et al.* [4] offer a more detailed look at various astrophysical contexts. Also worth a look are the books by Choudhuri [5] and Kulsrud [6], which have a greater emphasis on plasma effects, i.e. not using the single-fluid approximation.

## 2. Observed properties of magnetic stars

The observational techniques used for measuring magnetic fields on the surface of stars have been reviewed by Donati & Landstreet [7] and Mathys [8]. In almost all types of magnetic star, the Zeeman effect is used to detect the magnetic field. (For an excellent introduction of the Zeeman effect and its detection in stars see [9].)

The most reliable observations of magnetic stars use ‘full Stokes’ spectropolarimeters that record the complete information contained in linear and circular polarization. The full set of polarization components of a single suitable spectral line is sufficient in principle to determine the strength of the magnetic field and its orientation with respect to the line of sight. The V-signal, which gives information about the line-of-sight component of the magnetic field, is easier to detect than Q and U because it is antisymmetric with respect to the centre of the line and less sensitive to instrumental polarization.

To increase the signal-to-noise ratio of these measurements, many metal lines can be combined together to give a weighted mean of the Stokes I and V line profiles in a procedure known as least squares deconvolution (LSD; [10]). Since this technique was introduced, the detection limit has dropped significantly. What this gives us is a disc-averaged line-of-sight component of the magnetic field, the so-called *mean longitudinal field* *B*_*z*_; for many stars this is the only quantity that can be reliably measured. In other stars though it has also been possible to get extra information from Stokes Q and U; if the star is observed at several rotational epochs one can then construct a simple model of the magnetic field on the surface, e.g. dipole + quadrupole, and work backwards to find the best-fitting parameters of the model.

### 2.1. Ap stars

In this section, we review the Ap stars, the spectroscopically ’peculiar’ intermediate-mass main-sequence stars between about B8 and F0.

**Figure 1.**
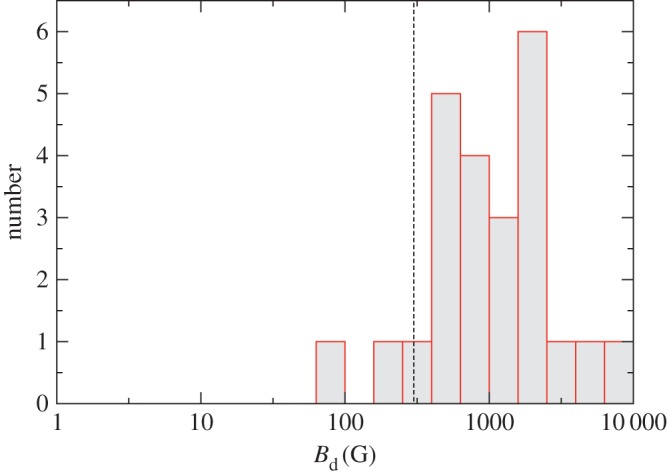
Measured magnetic fields in a sample of Ap stars in which either no magnetic field had yet been detected or in which there had been an ambiguous or borderline detection. In this study, the detection limit is only a few gauss; every star in the sample is found to have a magnetic field much stronger than this. The dashed line represents the cut-off at 300 G. This result confirms convincingly that all Ap-type chemically peculiar stars have strong magnetic fields. By contrast, other A stars have never been found to have any magnetic field above a few gauss; there is a clear bimodality. From Aurière *et al.* [11].

It has gradually become clear that there is a bimodality in the population of intermediate-mass stars (1.5 to 6*M*_⊙_), namely that all stars classified as Ap/Bp (with exception of the so-called mercury-manganese (HgMn) stars) host large-scale magnetic fields with mean longitudinal fields between around 200 G and 30 kG, and that the rest of the population lack magnetic fields above the detection limit of a few gauss ([11]; figure 1). Ap stars account for a few per cent of the population.

**Figure 2.**
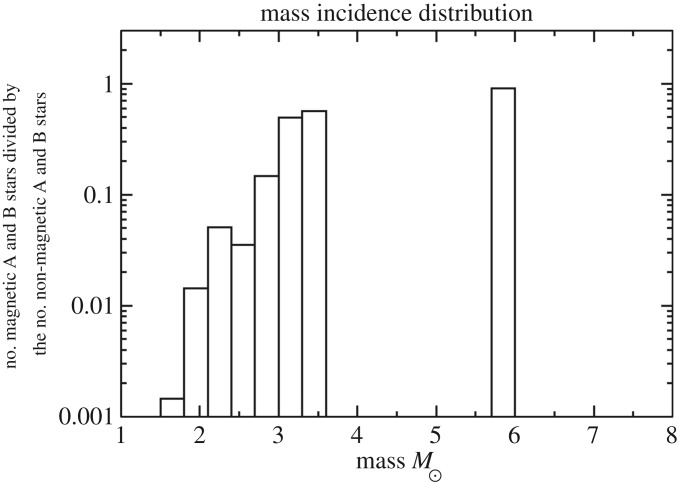
The fraction of intermediate-mass stars within 100 pc in which a magnetic field has been detected; the sample is relatively complete. From Power *et al.* [12].

Still, this leaves a factor of 100 in field strength to be explained by models for the origin of Ap star fields. A similar problem exists in the magnetic white dwarfs, where field strengths range from less than 10^4^ to almost 10^9^ G, and in pulsars (∼10^10^−10^15^ G). This problem may well reflect a basic property of the formation process of fossil fields (see also §4). There are various observational clues to the origin of this bimodality in magnetic properties among A and late B stars. For instance, there is a strong correlation between mass and the magnetic fraction of the population. Power *et al.* [12] examined a volume-limited sample of intermediate-mass stars—all stars within 100 pc of the Sun—finding that the magnetic fraction of the population increases from less than 1% at 1.5*M*_⊙_ to ≳20% at 3.5*M*_⊙_ (figure 2). The total magnetic fraction in the sample is only 1.7%. Note that the Ap phenomenon disappears completely at around F0, which coincides with the onset of efficient convection in the envelope (e.g. [13]). Other clues come from the rotation and binarity—see below.

In some strongly magnetized, slowly rotating Ap stars and most magnetic white dwarfs the Zeeman splitting is greater than the line width, in which case it is possible to measure the Zeeman splitting directly in Stokes I (intensity), without having to use polarimetry. This gives an average of the field strength over the visible disc, the so-called *mean field modulus* *B*_*s*_. Now, if a star’s magnetic field is dominated by small-scale structure we will clearly expect that *B*_*s*_≫|*B*_*z*_|, because the line-of-sight component from various patches on the surface will cancel each other in some kind of statistical $\sqrt{N}$ manner. For the Ap stars in which *B*_*s*_ is measurable, we do not find this—an important result showing that small-scale structure, if present, is not dominant.

Ap stars display a large variety of field geometries. In many stars, a good fit to the data can be achieved by assuming the simplest geometry of all, i.e. a dipole field, at the surface, which is inclined to the rotation axis at some angle. In other stars, this produces poor results and a more complicated geometry produces better results, for instance dipole + quadrupole.

**Figure 3.**
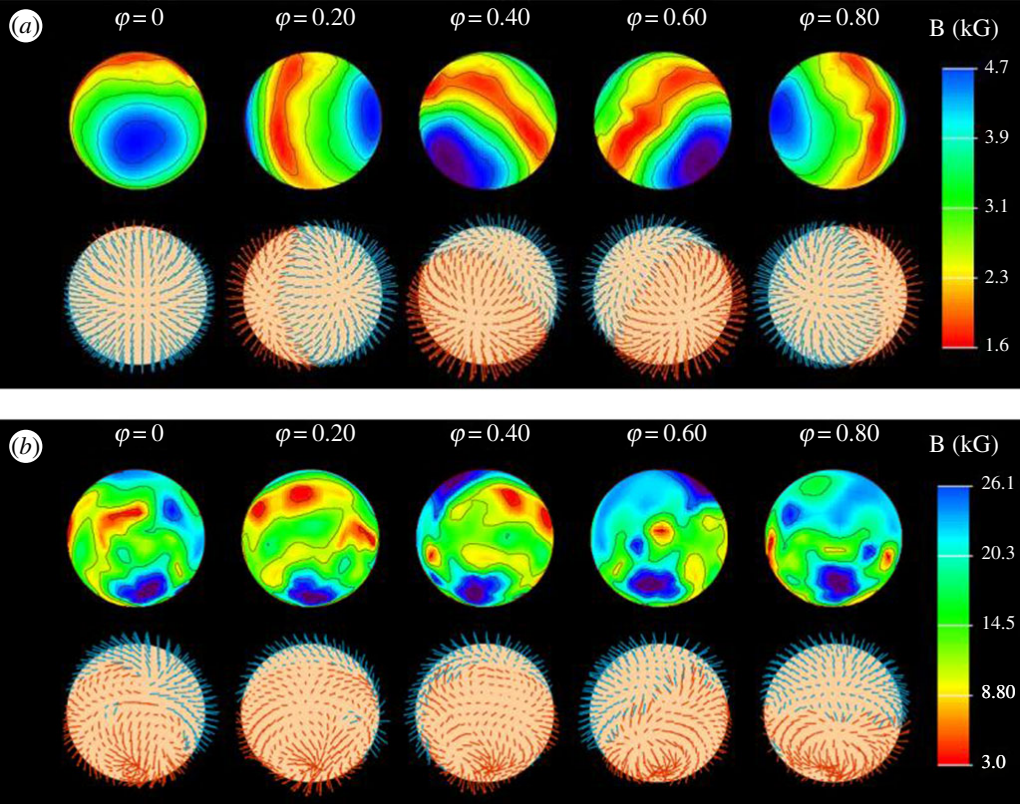
The magnetic fields of *α*^2^ CVn (*a*) and 53 Cam (*b*), viewed at five rotational phases. The upper rows in each panel show field strength and the lower rows the direction. Clearly, while *α*^2^ CVn has an almost perfect dipole field, the magnetic field of 53 Cam has a much more complex geometry. From Kochukhov *et al.* [15,16].

Improved observations have made it possible to combine the Zeeman effect with the Doppler effect from the rotation of the star to get, in effect, some spatial resolution on the surface of the star, without having to make prior assumptions of this kind. Piskunov & Kochukhov [14] have developed a technique called Magnetic-Doppler imaging and have used it to make some impressive maps of the magnetic field on a number of stars, such as 53 Cam [15], *α*^2^ CVn [16,17] and HD 37776 [18]. Two examples are shown in figure 3. A similar technique called Zeeman–Doppler imaging, developed by Donati and Petit (e.g. [19]) has been used to make magnetic images of cool stars, e.g. Petit *et al.* [20], as well as some hot stars, e.g. Donati *et al.* [21] and figure 7. Using these techniques, some rather complicated geometries^2^ have been found which appear to indicate the presence of meandering flux tubes just below the stellar surface.

#### 2.1.1. Chemical peculiarities

Interesting and unique to intermediate-mass stars are processes near the surface: gravitational settling and radiative levitation, which cause separation of chemical elements in the atmospheres of the stars and result in a variety of observed chemical abundance phenomena [22]. Ap/Bp stars are defined as a class showing peculiar (hence the ‘p’ in ‘Ap star’) abundances of rare earths and some lighter elements such as silicon, as well as inhomogeneities of these elements on the surface which show correlations with the magnetic field structure, albeit not the same kind of correlation in all stars. There is apparently a one-to-one correlation between the Ap/Bp phenomenon and strong magnetic field, with the apparent exception of the subclass of the HgMn stars [23].

The origin of this phenomenon is inextricably linked to the presence and location of surface and subsurface convection layers resulting from opacity bumps associated with helium and hydrogen ionization (see also §6.5). Convection obviously washes out the effects of any gentle separation processes, and a sufficiently strong magnetic field is expected to disrupt convection. A magnetic field ≳200 G is above equipartition with the thermal pressure at the photosphere, and should thus inhibit convection ([24–26], see also §6.5.2). Indeed, there is observational evidence for this in the form of a reduction in microturbulence velocities in Ap stars (D. Shulyak, personal communication). In spectral type, the Ap/Bp phenomenon disappears around F0, corresponding to the onset of efficient convection at the surface, and at B8, corresponding to the appearance of stronger subsurface convection.

The chemical peculiarities apparently develop after the magnetic field is already in place, appearing at some stage during the pre-main-sequence [27]. Note though that chemical peculiarities are not restricted to the magnetic stars; among the other A stars, various other types of chemical peculiarity are seen, for instance in the slowly rotating Am stars (of which Sirius is the best-known specimen), mercury-manganese stars and λ Bootis stars. See [28], for a review of these ‘skin diseases’.

#### 2.1.2. Rotation

It has long been known that most magnetic A stars rotate slowly compared with the non-magnetic stars (e.g. [29]). While the non-magnetic A stars are generally fast rotators, with rotation periods of a few hours to a day, most Ap stars have periods between 1 and 10 days, and some have periods much greater, with around 10% of Ap stars having periods above 100 days [30]. The slowest rotation periods are of the order of decades and in several cases only a lower limit can be stated. Note that while the rotation periods of the non-magnetic stars are estimated statistically from vsin⁡i, those of magnetic stars can be measured directly from the periodicity in the Zeeman signal, because the magnetic field is never perfectly symmetrical about the rotation axis.

**Figure 4.**
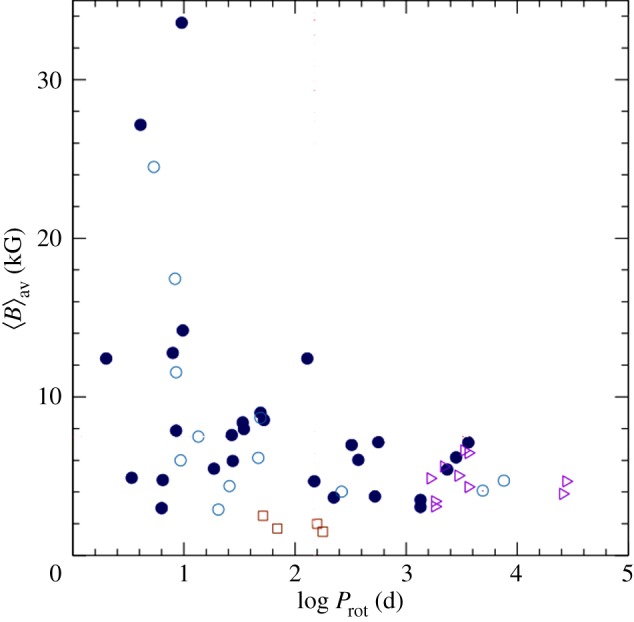
Observed average of the mean magnetic field modulus against rotation period. Dots: stars with known *P*_*rot*_; triangles: only a lower limit of *P*_*rot*_ is known. Open symbols: stars for which existing measurements do not cover the whole rotation cycle. From Mathys [31].

Some intriguing correlations between the rotation period and magnetic properties of Ap stars have been found. For instance, Mathys [30] finds in a sample of slowly rotating stars that those with *P*>100 days lack fields in excess of 7.5 kG. A recent compilation is shown in figure 4. In addition, Landstreet & Mathys [32] found that the slower rotators (*P*>25 days) are more likely to have closely aligned magnetic and rotation axes.

The slow rotation of Ap stars holds only in a very broad sense; rapidly rotating examples like CU Virginis (0.5 d) also exist. Any given explanation for the slow average rotation may well miss the most important clue: the astonishingly large range in rotation periods, of at least four orders of magnitude.

#### 2.1.3. Binarity

The binary fraction among the magnetic stars is lower than in the non-magnetic stars [33–36]. There is apparently a complete lack of Ap stars in binaries with periods of less than about 3 days, except for one known example (HD 200405) with a period of 1.6 days. This speaks in favour of more than one of the formation scenarios (see §4).

#### 2.1.4. Ages

Pre-MS stars are known as T Tauri stars if they are later than spectral type F5 (log⁡T≈3.8) and Herbig Ae-Be (HAeBe) if they are earlier. We now know of over 100 HAeBe stars (e.g. [37,38]). Stars between about 1.5 and 4*M*_⊙_ leave the birth line as fully convective T Tauri stars. Eventually, they develop a radiative core, at which time they stop moving downwards on the HR diagram and move instead to the left on what is known as the Henyey track; the convective envelope shrinks and they become HAeBe stars. More massive stars leave the birth line as HAeBe stars. Depending on the local and/or accretion conditions as well as on the mass, these stars may or may not become visible before they reach the MS; the most massive HAeBe stars observed are around 20*M*_⊙_.

**Figure 5.**
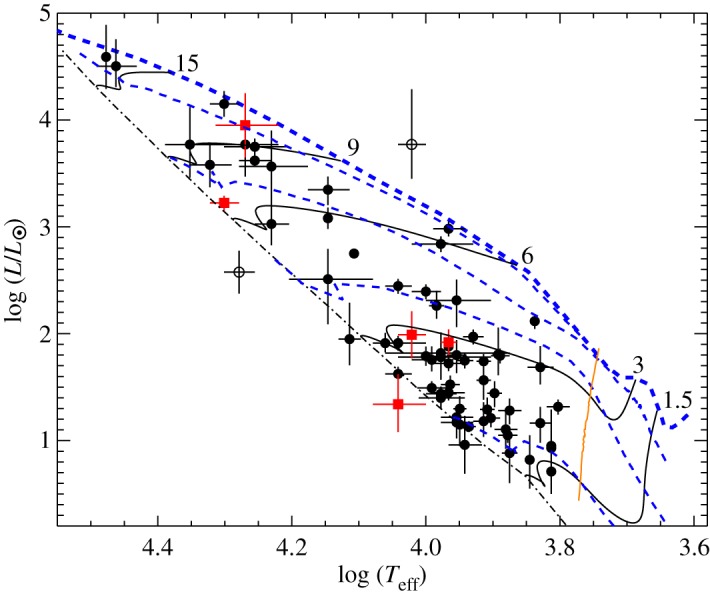
Magnetic (red squares) and non-magnetic (black circles) HAeBe stars on an H-R diagram. The thick blue dashed line is the birth line, the thin blue dashed lines are isochrones, the dot-dashed line is the ZAMS and the solid lines are theoretical evolutionary tracks from the birth line to the ZAMS for the masses indicated. The orange line represents a radiative/convective transition: to the left of this line, the convective envelope accounts for less than 1% of the star’s mass. A convective core appears towards the end of the PMS when the star moves downwards on the HR diagram. The open circles correspond to HD 98922 (above the birth line) and IL Cep (below the ZAMS), whose positions cannot be reproduced with the theoretical evolutionary tracks considered. From fig. 4 in Alecian *et al.* [40].

It is clear now that some fraction—comparable with the fraction among main-sequence A and B stars—of HAeBe stars are magnetic. Wade *et al.* [39] presented the first detections of magnetic fields in HAeBe stars, and Alecian *et al.* [40,41] present the results from a survey of 70 Herbig Ae-Be stars (figure 5).

There was a claim [42] that all Ap stars have passed through at least 30% of their main-sequence lifetime. This result depended on determining the ages of stars by placing them on the HR diagram, which is very challenging because stars move very slowly across the HR diagram during the first part of the main sequence, and because of the distortion of apparent surface temperatures by the atmospheric abundance anomalies. In the light of this, and of the recent results on pre-MS stars, it looks unlikely that this result is correct. Landstreet *et al.* [43] looked instead at Ap stars in clusters—where the ages can be determined much more accurately—and found the opposite: that there is a negative correlation between the field strength in Ap stars and their age, greater than one would expect from flux conservation as the star expands along the main sequence. Possible explanations for this field decay are discussed in §3.8.

#### 2.1.5. Variability in Ap stars

**Figure 6.**
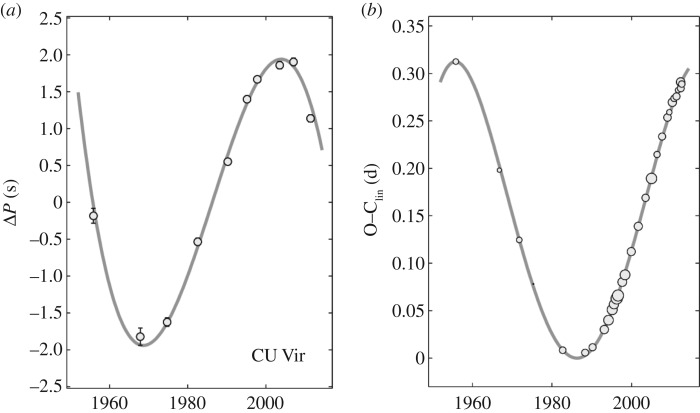
Variation in the rotation period of CU Virginis. Panel (*a*) shows the variation in the rotation period, and panel (*b*) is the phase residue, assuming a constant period. From Mikulášek *et al.* [45].

Though Ap stars are characterized by stability of their magnetic and spectral signatures on all observable timescales (apart from rotational modulation), there exist a few curious cases where changes have been observed. The rapidly rotating star CU Virginis (*P*=0.52 days, with observations stretching back to 1949 [44]), has increased its rotation period by about 50 ppm (figure 6). The O–C data (the phase drift relative to a fixed period) can be fit by an increase in rotation period within a few years around 1984 [46,47]^3^). This would be reminiscent of the ‘glitches’ seen in pulsars. However, a more gradual change in period also seems compatible with the observations. Stȩpień [48] suggested that the changes in CU Vir may not actually be monotonic, but could reflect some form of magnetic oscillation in the star, with typical timescales of several decades. This would imply that, at a given point in time, one would find period decreases about as frequently as increases. Period increases have been reported in other stars: spindown rates of the order of $P/\dot{P}∼10^{5}-10^{6}\;{\mathrm{yr}}^{-1}$ have also been reported in V901 Ori, *σ* Ori E, HR 7355, SX Ari and EE Dra [49]. No clear case of a period decrease has so far been seen in the sample of stars with changing periods, but there are hints that both CU Virginis and V901 Orionis may now be spinning up [45]. The current limited sample looks therefore still to be statistically compatible with the idea; finding alternative explanations would be challenging. Speaking strongly in favour of the oscillation idea is that the spindown timescales measured are of the order of 10^5^−10^6^ yr, which would otherwise be hard to reconcile with the ages inferred for these stars, of the order of 10^8^ yr.

Mikulášek *et al.* [50] offer two alternative interpretations of the data on CU Virginis, namely that the rotation period is undergoing either some kind of Gaussian variation and will return to its original value, or a periodic variation with a minimum rotation period in 1968 and a maximum in 2005. In the oscillation scenario, the amplitude (at the surface) of the torsional oscillation phase residue in CU Vir would be approximately 2.3*π* and in V901 Ori at least 0.5*π* [50]. A challenge for the oscillation idea might be that the Alfvén timescale in CU Vir, with a surface field strength of around 3 kG, would be only about 3 years, assuming that field strength also in the star’s interior. One expects the fundamental torsional oscillation mode to have this period, and higher harmonics even shorter periods.

A remarkably rapid change in the field configuration, on a timescale of years, was reported in the pre-MS star HD 190073 [51]. It now appears that this was a spurious result [52].

### 2.2. Other stably stratified stars

Having looked in some detail at the strongly magnetic subset of intermediate-mass stars, which have a long history in the literature, we now turn to other stars which are stably stratified, at least on the outside. First of all, the rest of the intermediate-mass population and the more massive stars. We look then briefly at white dwarfs. Neutron stars are discussed separately in §7.

#### 2.2.1. Vega and Sirius

As far as the ‘non-magnetic’ part of the population of intermediate-mass stars is concerned, an exciting discovery has been the detection of magnetic fields in Vega and Sirius, the two brightest A stars in the sky. Zeeman polarimetric observations of these two stars have revealed weak magnetic fields: in Vega a field of 0.6±0.3 G [53,54] and in Sirius 0.2±0.1 G [55]. The field geometry is poorly constrained, except that the field should be structured on reasonably large length scales, as cancellation effects would prevent detection of a very small-scale field. Vega seems to have a strong (∼3 G) magnetic feature at its rotational pole. The existence or otherwise of time variability is unknown—Petit *et al.* simply note that in Vega ‘no significant variability in the field structure is observed over a time span of one year’ [54]. These two stars have similar mass but have significant differences: Vega is a rapidly rotating single star, and Sirius is a slowly rotating Am star which may well have accreted material from its companion [56]. Given that we so far have detections in both stars observed, it seems very likely that the rest of the ‘non-magnetic’ population also have magnetic fields of this kind. There are also theoretical grounds to expect this (see §3.7).

#### 2.2.2. Massive main-sequence stars

Direct detection via the Zeeman effect of magnetic fields in stars above around 6 or 8*M*_⊙_ is significantly more challenging than that in intermediate-mass stars. Firstly, because there are fewer lines in the spectrum, and as the signals from each line are normally added together with the LSD technique (see above), this leads to a smaller signal. Secondly, because there are various line-broadening mechanisms. While the atmospheres of A and late B stars are very quiet, O and early B stars display a number of observational phenomena such as discrete absorption components, line profile variability, wind clumping, solar-like oscillations, red noise, photometric variability and X-ray emission. Much of this has to do with winds and wind variability, and much is not yet understood (e.g. [57] and references therein). This complicates the life of the Zeeman observer, with the result that the detection limit on magnetic fields is perhaps 30 or 100 G, much higher than in A stars. See for instance Henrichs [58], for a recent review of magnetism in massive stars.

**Figure 7.**
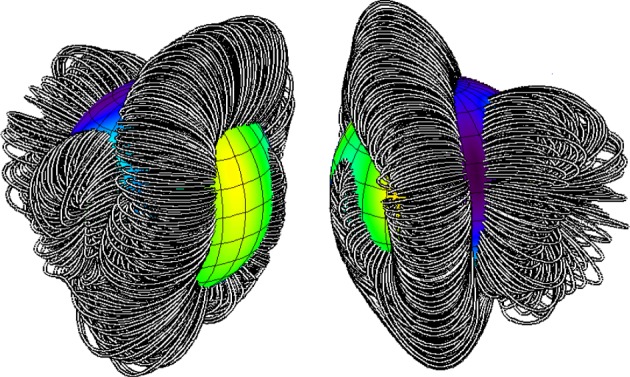
The observed field geometry on the main-sequence B0 star *τ* Sco, at two rotational phases, using Zeeman-Doppler imaging. The paths of arched magnetic field lines are clearly visible; it seems likely that they are associated with twisted flux tubes buried just below the surface—see §3.5. From Donati *et al.* [21]. Observations of this star taken several years apart show the same topology, confirming a lack of variability also in stars with complex magnetic topologies [7].

The result of this is that magnetic fields were not detected in hot stars until relatively recently [59], but with the completion of recent surveys (e.g. the MiMeS survey, [60]) we have a much better picture of the incidence of large-scale magnetic fields in massive stars. It seems that around 7% of the population host large-scale fields [61]. The magnetic stars have fields of 300 G–10 kG, and a variety of geometries, as is found in the A stars. While some of the magnetic stars have an approximately dipolar field, others are found to have a more complicated geometry (see figure 7, for an example). In several other stars, similarly complex magnetic fields have been found, dubbed the ‘*τ* Sco clones’. Recently, it has been found that the magnetic flux in magnetic OB stars decays during the main sequence, the same as is found in Ap stars (§2.1.4). Possible explanations are discussed in §3.8.

It is tempting to conclude, therefore, that the phenomenon is simply a continuation of that seen in intermediate-mass stars, and indeed there are no particular theoretical reasons to think otherwise. The historical division between magnetism in intermediate- and high-mass stars is probably due only to the observational difficulty of observing the Zeeman effect in the hotter stars, and perhaps more importantly the fact that magnetic fields in hotter stars do not give rise to chemical peculiarities as seen in intermediate-mass stars, making them harder to identify. The reason for the lack of such peculiar abundances is presumably that the wind removes the outer layers before chemical-separating mechanisms have time to work.

The ‘non-magnetic’ majority of the population may well have weaker and/or smaller-scale magnetic fields of some kind, and the various observational phenomena mentioned above could plausibly be at least partly the result of magnetic activity, not unlike that in the Sun—see §6.5.

#### 2.2.3. White dwarfs

The stable fields observed in the subclass of magnetic white dwarfs (mWD) show parallels with those of Ap stars, though it is not clear if this is more than a coincidence. The subclass is similarly small, the range in field strength again large, ranging from a few times 10^3^ to 5×10^8^ G. As in the case of Ap stars, the large range in rotation periods of mWD is puzzling. Most rotate at periods from a fraction of a day to several weeks, but some of them have inferred periods as long as decades. For more on their properties, we refer to reviews by Putney [62] and Schmidt [63].

White dwarfs have been important for the theory of fossil fields because, unlike early-type main-sequence stars, they do not contain any significant convective zones. Consequently, as far as the nature of their magnetic fields is concerned, there is essentially no alternative to a magnetic equilibrium. They are the same in this respect as the neutron stars, to which §7 is devoted; indeed much of the physical processes discussed in that section also applies to white dwarfs. In §3.1, we discuss magnetic white dwarfs in the context of the origin of fossil fields in general. They also offer clues regarding the rotation of the cores of giant stars and angular momentum transport within stars in general (see §5.5).

## 3. Theory of fossil fields

In radiative stars, the main challenges are to explain not just how these stars come to host magnetic fields at all, but to explain the large range in the magnetic and rotational properties of otherwise similar stars. There are similarities in these respects between Ap stars, magnetic massive stars, magnetic white dwarfs (mWD) and to some extent also the neutron stars.

An early idea triggered by these similarities, still widely referred to, is that of ‘flux freezing’. It does not attempt to explain the fields of Ap stars themselves, which are just taken as an observational given. Instead, it interprets the magnetic flux of white dwarfs and neutron stars as ‘inherited’ from magnetic main-sequence progenitors, the Ap, Bp and magnetic O stars. Ideas for the origin of these progenitors themselves are even less well developed. An obvious connection that must play a role is that with the magnetic fields observed in protostellar clouds, but this in itself does not tell us what to make of the puzzling range of field strengths in Ap stars, their low flux-to-mass ratio and overall low frequency. This is discussed in more detail in §4.

### 3.1. Flux freezing

The large field strengths of mWD can be understood as a consequence of the greater compactness of WD compared with main-sequence stars. An mWD may have a radius of ∼5×10^8^ cm, a factor ∼100 smaller than the core of a mid-A main-sequence star. A popular hypothesis is that mWD inherited their fields from Ap progenitors. Under the assumption of ‘flux freezing’, the magnetic field lines would stay anchored in the star during its evolution through the giant branch, the planetary nebula (PN) phase and the evolution of the remaining core to a WD. This would predict the field strength of an mWD to be ∼100^2^ higher than the field of the MS stars it started with, which would put it in the observed range.

The flux inheritance hypothesis is nevertheless somewhat dubious, as it implies that the loss of more than half of the star’s mass in the PN phase does not significantly affect its magnetic flux. Figures 9 and 12 do suggest that some fraction of the surface flux of the Ap star may possibly pass through the core that ends up forming the WD. This flux consists, however, of poloidal field lines. The subsurface torus surrounding this poloidal flux is what maintains the stability of the configuration. It would be lost during the ejection of the envelope in the PN phase. The remaining poloidal configuration would be highly unstable via the Flowers–Ruderman mechanism (§3.3). Numerical results of this process suggest that not much of the poloidal field would survive from such a configuration.

Another way of looking at the field strengths is by comparing magnetic energy density (*B*^2^/8*π*) with other energy densities in the star. For a magnetic star to be bound, its magnetic energy must be less than the gravitational binding energy. Per unit mass, this is of the order of the central pressure *p*_*c*_. The central pressure in stars of mass *M* and radius *R* scales as *M*^2^/*R*^4^ (e.g. [64]). Comparing the two predicts a scaling of the maximum possible field strength, *B*_max_∼*M*/*R*^2^. In terms of the magnetic flux: *Φ*_max_∼*B*_max_*R*^2^∼*M*. As the mass of a WD is only a few times smaller than an Ap star, the similarity of the maximum magnetic flux in the two cases is effectively the same as predicted by ‘flux freezing’. Magnetic field strengths from a flux freezing argument can therefore not be distinguished from arguments relating field strength to energy density (which imply very different physics).

The maximum values of the surface field strength observed are of the order of 2×10^4^, 5×10^8^ and 10^15^ G for Ap stars, mWD and neutron stars, respectively. These numbers are a factor 10^4^–10^3^ smaller, respectively, than the maximum field strengths allowed by equipartition with the gravitational binding energy of the stars. It is not clear if this factor, or its similarity between the different types of star, has a special meaning. In any case, these maxima do not constrain theories for the origin of the field very much.

### 3.2. The nature of magnetic fields in radiative stars

Since the discovery of magnetic fields in Ap stars, there have been two theories to explain their presence: the core-dynamo theory and the fossil field theory. According to the former, the convective core of the star hosts a dynamo of some kind, which sheds magnetic field into the overlying radiative layer. This magnetic field then rises, under the action of buoyancy, towards the surface. The reason for this buoyant rise can be understood as follows. A magnetic feature (e.g. a flux tube) must be in total pressure equilibrium with its non-magnetized surroundings: the sum of gas, radiation and magnetic pressures inside the feature must be equal to the sum of gas and radiation pressures in the surroundings. To prevent buoyant rise, the density of the feature must be the same as that of the surroundings, which is only possible if the temperature is lower. This causes heat to diffuse into the magnetic feature, causing it gradually to rise. However, it turns out that the timescale for this buoyancy process is longer than the main-sequence lifetimes of these stars unless very small flux tubes can be generated [65–67]. This does not agree with the observations, which suggest mainly large-scale structure at the surface. In addition, the retreating convective core (in mass coordinates) leaves behind a strong chemical gradient in the radiative layers, enormously slowing down the escape process [68]. Also puzzling in this theory is the enormous range of field strengths between different stars which are predicted to have similar convective cores.

The fossil field theory, on the other hand, appears better able to explain the observations, in particular the large-scale geometry, and large field strengths. The basic idea is that instead of being continually regenerated in some ongoing dynamo process feeding off the star’s luminosity, the field is in a stable equilibrium in a static radiative zone. In MHD, the magnetic field **B** evolves according to the induction equation

3.1 $$\frac{\partial \text{B}}{\partial t}=\nabla \times (\text{u}\times \text{B}-\eta \nabla \times \text{B}),$$

where **u** is the fluid velocity and *η* is the magnetic diffusivity, the reciprocal of the electrical conductivity. In turn, the velocity field is related to the forces acting on the gas, i.e. the pressure gradient, gravity and Lorentz forces via the momentum equation

3.2 $$\frac{d\text{u}}{dt}=-\frac{1}{\rho }\nabla P+\text{g}+\frac{1}{4\pi \rho }(\nabla \times \text{B})\times \text{B},$$

where *P*, *ρ* and **g** are pressure, density and gravity, respectively. In an equilibrium unmagnetized star, the pressure gradient and gravity balance each other. Upon the addition of an arbitrary magnetic field, the Lorentz force will cause the gas to move at approximately the Alfvén speed $v_{A}=B/\sqrt{4\pi \rho }$, and the system evolves on an Alfvén timescale *τ*_A_=*R*/*v*_A_ (where *R* is the radius of the star) which in a star with a 1 kG field is around 10 years. Eventually, one might hope to reach an equilibrium—a so-called fossil field—where the three forces balance each other and **u**=**0**, so that the first term on the r.h.s. of the induction equation disappears and the field evolves only on a diffusive timescale *R*^2^/*η*. Cowling [69] first realized that this timescale is of the order of 10^10^ years in the radiative core of the Sun and that a field in equilibrium there could therefore persist for the entire lifetime of the star. The magnetic equilibrium must also be stable, however, because instability timescales are of the order of the Alfvén timescale, which as mentioned above can be as short as a few years.

Much effort has been put into finding such stable equilibria, which was historically also motivated by the need for magnetic plasma confinement in nuclear fusion devices. Unfortunately, it turns out to be a difficult problem to solve, and with analytic techniques the existence of these stable equilibria was never convincingly demonstrated. This was historically a major weakness of the fossil field theory but, in the light of the weaknesses of the core dynamo theory (including the discovery of magnetic fields with similar properties in white dwarfs, which contain no convective core), there was a widespread feeling that stable equilibria must exist.

Using analytic techniques, attempts to find such equilibria are split into two parts: first finding an equilibrium, and then checking its stability. The first step should not, prima facie, represent any major problem. To see this (without an actual proof), consider the following. The magnetic field has two degrees of freedom (reduced from three by the **∇**⋅**B**=0 constraint) and so the Lorentz force should also have two degrees of freedom. Ignoring thermal diffusion, the thermodynamic state of the gas also has two degrees of freedom. Where the magnetic field is weak—in the sense that the plasma-*β*=8*πP*/*B*^2^≫1—equilibrium can be obtained by suitable adjustments of the thermodynamic variables, say pressure and entropy.^4^ Where the field strength is not small in this sense, for instance close to or above the surface of a star, an equilibrium must be close to a force-free configuration. We can therefore divide the star conceptually into two domains: the interior of the star where *β*≫1 and the allowed range of field geometries is not strongly constrained (as long as equilibrium is the only concern and stability is ignored), and the exterior where *β*≪1 and the field is close to force-free. Near the photosphere of the star the gas pressure scale height is much smaller than the length scales on which the magnetic field changes, and so for any Ap star the *β*=1 surface will be fairly close to the photosphere. In fact if the field strength at the photosphere is 300 G, as in the weakest-field Ap stars, then *β*=1 coincides with the photosphere; in Ap stars with stronger fields this surface is a little lower down.

In addition to the MHD processes above, field generation by microscopic processes have been considered: the ‘Biermann battery’ ([70] cf. [6]) and the thermoelectric effect (Dolginov & Urpin [71], Urpin & Yakovlev [72]). In the stellar environment, both operate very slowly, as they depend on diffusion. For them to be effective, a very stable environment is required. In an A star, such an environment can be supplied by a stable fossil field, but appealing to this would obviously cause circular reasoning. The situation is better for neutron star crusts, where its solid state can provide a stable environment there. It has been concluded that the process is unlikely to be effective in this case, however, because the field produced in this way early in the life of the neutron star would decay when it cools Blandford *et al.* [73].

### 3.3. Stability of fossil equilibria

To check the *stability* of an equilibrium using analytic techniques is rather trickier. Most studies use the energy method of Bernstein *et al.* [74] where the energy change in a configuration is calculated as a displacement perturbation is applied. If the energy change is positive for all possible perturbation fields, the configuration is stable. In the stellar context, this method was more successful in uncovering instabilities than in demonstrating stability. For reasons of simplicity, almost all effort has been concentrated on axisymmetric equilibria. Tayler ([75], see also [76]) looked at purely toroidal fields, that is, fields that have only an azimuthal component *B*_*ϕ*_ in some spherical coordinate frame (*r*,*θ*,*ϕ*) with the origin at the centre of the star. He derived necessary and sufficient stability conditions for adiabatic conditions (no viscosity, thermal diffusion or magnetic diffusion). The main conclusion was that such purely toroidal fields are always unstable to adiabatic perturbations at some place in the star, in particular to perturbations of the *m*=1 form (*m* being the azimuthal wavenumber). Numerical simulations have also been used to look at the stability of purely toroidal fields [77], reproducing many of the analytic results.

**Figure 8.**
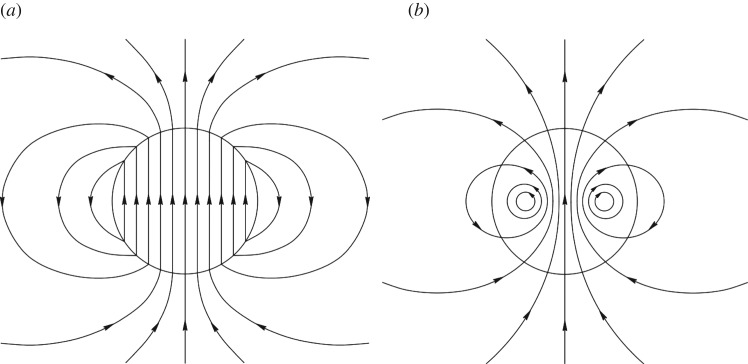
Poloidal field configurations. (*a*) All field lines close outside the star, this field is unstable by an argument due to Flowers & Ruderman [78]. For the case where some field lines are closed inside the star, instability was demonstrated by Markey & Tayler [79,80] and Wright [81].

The opposite case is a field in which all field lines are in meridional planes (*B*_*ϕ*_=0; figure 8). In subsequent papers, Markey & Tayler [79,80] and, independently, Wright [81] studied the stability of axisymmetric poloidal fields in which (at least some) field lines are closed within the star (figure 8*b*). These fields were again found to be unstable.

A case not covered by these analyses was that of a poloidal field in which *none* of the field lines close within the star. An example of such a field is that of a uniform field inside, matched by a dipole field in the vacuum outside the star (figure 8*a*). This case has been considered earlier by Flowers & Ruderman [78], who found it to be unstable by the following argument. Consider what would happen to such a dipolar field if one were to cut the star in half (along a plane parallel to the magnetic axis), rotate one half by 180^°^, and put the two halves back together again. The magnetic energy inside the star would be unchanged, but in the atmosphere, where the field can be approximated by a potential field, i.e. with no current, the magnetic energy will be lower than before. This process can be repeated *ad infinitum*—the magnetic energy outside the star approaches zero and the sign of the field in the interior changes between thinner and thinner slices. Marchant *et al.* [82] present a more rigorous analysis of this instability. The reduction of the external field energy is responsible for driving the instability. As the initial external field energy is of the same order as the field energy inside the star, the initial growth time of the instability is of the order of the Alfvén travel time through the star, as in the cases studied by Markey & Tayler and Wright.

Prendergast [83] showed that an equilibrium can be constructed from a linked poloidal–toroidal field, but stopped short of demonstrating that this field could be stable. As both purely toroidal fields and purely poloidal fields are unstable, an axisymmetric stable field configuration, if one exists, should presumably be such a linked poloidal–toroidal shape. Wright [81] showed that a poloidal field could be stabilized by adding to it a toroidal field of comparable strength. However, the result was again somewhat short of a proof.

These results were all valid only in the absence of dissipative effects and rotation. The only case in which dissipative effects have been investigated in detail is that of a purely toroidal field (Acheson [84], Hughes & Weiss [85]), where it is found, for instance, that some combinations of diffusivities can have a destabilizing effect on configurations which are stable to the non-diffusive Parker instability. These effects have still to be investigated in a more general geometry such as that of the mixed poloidal–toroidal equilibria.

The effect of rotation was investigated by Pitts & Tayler [86] for the adiabatic case (i.e. without the effects of viscosity, magnetic and thermal diffusion). These authors reached the conclusion that although some instabilities could be inhibited by sufficiently rapid rotation, other instabilities were likely to remain, whose growth could only be slowed by rotation—the growth timescale would still be very short compared with a star’s lifetime. Also, diffusion can reduce the stabilizing effect of rotation [87]. Importantly though, rotation does not introduce any new instabilities: a configuration which is stable in a non-rotating star should also be stable in a rotating star.

### 3.4. Numerical results

More recently, it has become possible to find stable equilibria using numerical methods. Various equilibria are found. The simplest equilibrium consists of a single flux tube around the magnetic equator of the star, surrounded by a region of poloidal field. More complex equilibria (§3.5) can have more than one tube in various arrangements. From observations of magnetic A, B and O stars, we see that both the simple and the more complex equilibria do occur in nature.

**Figure 9.**
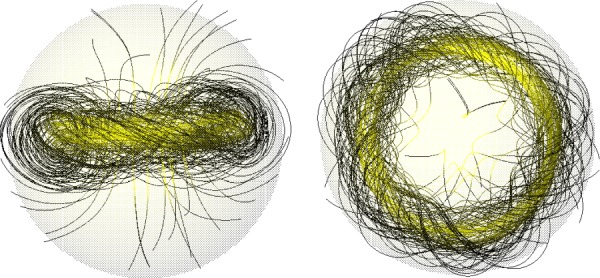
The shape of the stable twisted-torus field in a star, viewed along and normal to the axis of symmetry. The transparent surface represents the surface of the star; strong magnetic field is shown with yellow field lines, weak with black. Figure from Braithwaite & Nordlund [89].

Essentially, the numerical method consists in evolving the MHD equations in a star containing initially some arbitrary field. Braithwaite & Spruit [88] and Braithwaite & Nordlund [89] modelled a simplified radiative star: a self-gravitating ball of gas with an ideal gas equation of state, ratio of specific heats $\gamma =\frac{5}{3}$ and a stratification of pressure and density as in a polytrope of index *n*=3, embedded in an atmosphere with low electrical conductivity. Over a few Alfvén timescales, the field organizes itself into a roughly axisymmetric equilibrium with both toroidal and poloidal components in a twisted-torus configuration, illustrated in figure 9. This corresponds qualitatively to equilibria suggested by Prendergast [83] and Wright [81].

The stability of these axisymmetric fields, and in particular the range of possible ratios of toroidal to poloidal field strength, was examined further by Braithwaite [90] with a mixture of analytic and numerical methods. It was found that the fraction of energy in the poloidal component must satisfy $a(E/E_{\mathrm{grav}})<E_{p}/E≲0.8$ where *E* and *E*_grav_ are the total magnetic energy and the gravitational energy, *E*_p_ is the energy of the poloidal field and *a* is some dimensionless factor of the order of unity. Akgün *et al.* [91] got the same results with more analytic methods. To give some numbers, for an A star the dimensionless factor *a*∼15 and the ratio *E*/*E*_grav_ is only about 10^−6^ even in the most strongly magnetic Ap star (*B*≈30 kG), so that in this star we require for stability $10^{-5}≲E_{p}/E≲0.8$. In a neutron star *a*∼400 [91], and assuming a magnetar field strength of 10^15^ G, the condition is $4\times 10^{-4}≲E_{p}/E≲0.8$. In stars with weaker fields of course, the lower limit to the ratio *E*_p_/*E* is even lower.

Physically, the upper limit comes from the need for a comparable-strength toroidal field to stabilize the instability of a purely poloidal field, and is in rough agreement with the result of Wright [81]. The lower limit is different because the instability of a purely toroidal field, unlike that of a poloidal field, involves radial motion and so the stable entropy stratification has a stabilizing effect, preferentially on the longer wavelengths which involve greater radial motion. The poloidal field stabilizes preferentially the shorter wavelengths, and at sufficient poloidal field strength the two effects meet in the middle and all wavelengths are stabilized. The stable stratification is more effective if the field is weaker, hence the presence of the total field energy in the threshold. As *E*/*E*_grav_ is always a very small number, only a relatively small poloidal field is required. See Braithwaite [90] for a more thorough explanation.

This result is of particular interest in the context of neutron stars, where the deformation of the star from a spherical shape depends crucially on this ratio *E*_p_/*E* because poloidal field makes the star oblate and toroidal field prolate. In the presence of a suitable mechanism to damp torque-free precession, a prolate star should ‘flip over’ until the magnetic and rotation axes are perpendicular, which is the state in which the rotational kinetic energy is at a minimum, given a constant angular momentum. In this state, the star emits gravitational waves (e.g. [92]).

### 3.5. Non-axisymmetric equilibria

**Figure 10.**
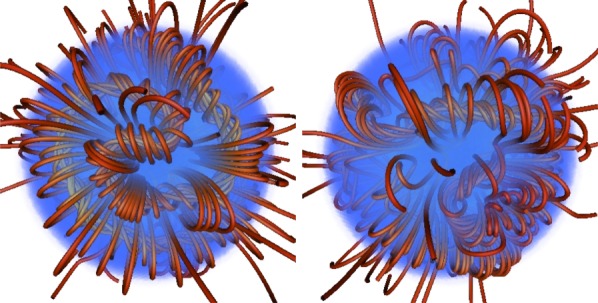
A typical non-axisymmetric equilibrium as found in simulations, viewed from both sides of the star. This corresponds qualitatively to those observed on stars such as *τ* Sco (see figure 7). Figure from Braithwaite [93].

**Figure 11.**
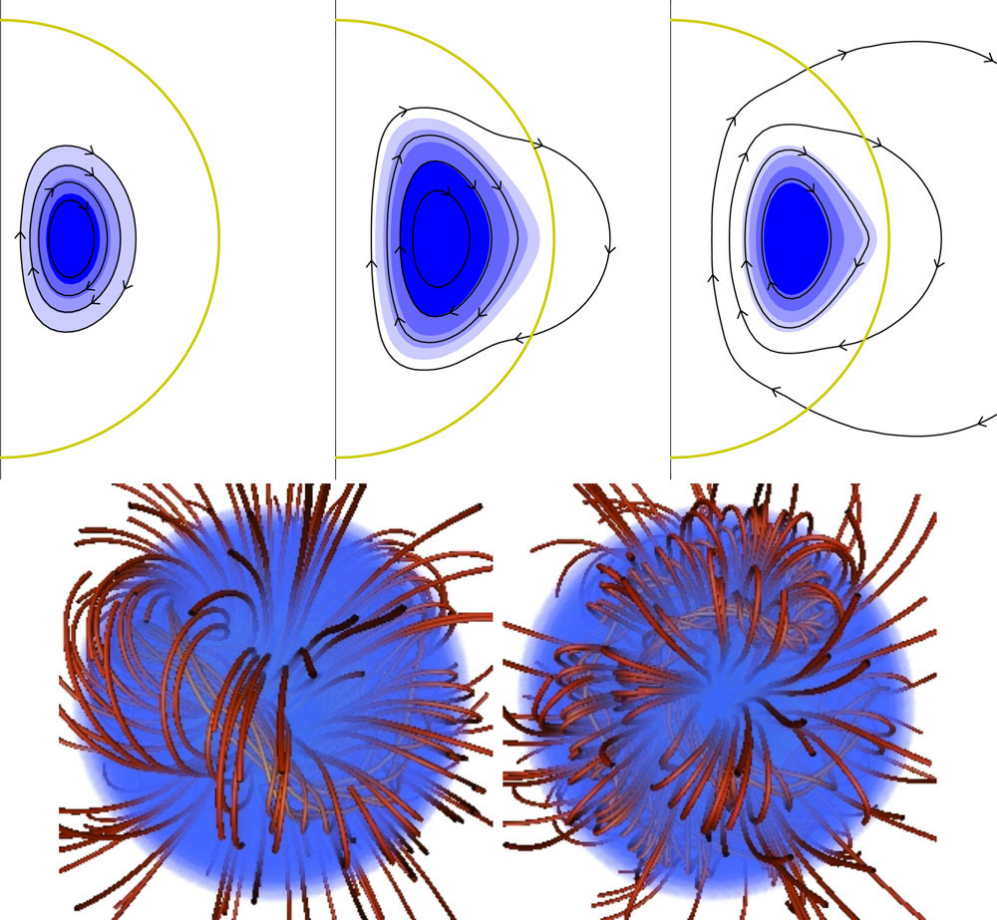
A sequence of equilibria resulting from initial conditions with different degrees of central concentration of the magnetic energy—above left, centrally concentrated initial conditions; above right, medium concentration (still leading to a roughlyaxisymmetric equilibrium); and below, flatter distributions; below right the radial energy distribution is completely flat. For the three axisymmetric equilibria, the (relatively small) non-axisymmetric component is ignored: plotted are contours of the flux function of the poloidal field, which are also poloidal field lines, and the shading represents the toroidal field multiplied by the cylindrical radius. For the two non-axisymmetric equilibria shown, field lines are plotted in red and the surface of the star is shaded blue. Note that these two equilibria have flux tube(s) of different widths (corresponding to the angle *α* in figure 12). Figures from Braithwaite [90,93].

From further simulations [93], it became clear that depending on the initial conditions, a wide range of equilibria can form, including non-axisymmetric equilibria (figure 10). Crucial is the distribution of magnetic energy and the amount of flux passing through the stellar surface to the low-conductivity medium outside. It is important to note that during relaxation to equilibrium there is essentially no radial transport of gas and magnetic flux, fluid motion being confined to spherical shells, so the total unsigned flux through any spherical shell ∮|B⋅dS| can only fall. Therefore, an initial field which is buried in the interior of a radiative star or zone evolves into a similarly buried equilibrium. It turns out that in this case, an approximately axisymmetric field forms. At the opposite end of the spectrum, an initial field with a flat radial field-strength profile with a finite amount of magnetic flux at the surface evolves into a non-axisymmetric equilibrium (figure 11). It seems that both axisymmetric and non-axisymmetric equilibria do form in nature, and both types can be found among A, B and O main-sequence stars as well as among white dwarfs (figures 3 and 7).

**Figure 12.**
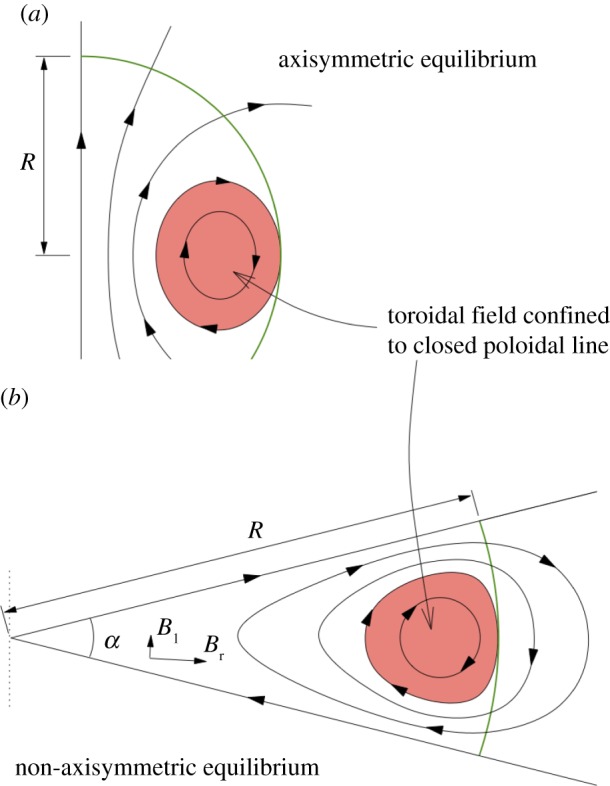
Cross-sections of magnetic equilibria, both of which contain a twisted flux tube surrounded by a volume containing just poloidal field. The stellar surface is shown in green and poloidal field lines (in black) are marked with arrows. The toroidal field(direction into/out of the paper, red shaded area) is confined to the poloidal lines which are closed within the star. (Toroidal field outside this area would unwind rather like a twisted elastic band that is not held at the ends.) (*a*) The axisymmetric case where the flux tube lies in a circle around the magnetic equator (corresponding to figure 9). (*b*) A narrower flux tube (corresponding to figure 10). In this case, the flux tube must be longer than in the axisymmetric case in order to occupy the whole stellar volume; it meanders around the star in an apparently random fashion, and there may also be two or more such tubes. Figure from Braithwaite [93].

The geometries of these various equilibria have one feature in particular in common, namely that they can be thought of in terms of twisted flux tubes surrounded by regions of purely poloidal field. The simple axisymmetric equilibrium can be thought of as a single twisted tube wrapped in a circle (a ‘twisted torus’); passing through this circle are poloidal field lines which pass through the stellar surface. The more complex equilibria contain one or more twisted flux tubes meandering around the star in apparently random patterns; these do not touch each other but are surrounded by regions where the field is perpendicular to the flux-tube axis. In the equilibria found thus far, the meandering is done at roughly constant radius a little below the surface. Equilibria where the flux tubes do not lie at constant radius seem plausible but it also seems plausible that they are difficult to reach from realistic initial conditions, especially in view of the restriction of motion to spherical shells. Figure 12 shows cross-sections of both the axisymmetric and more complex equilibria. The toroidal field is confined to the region where the poloidal field lines are closed within the star—this region resembles a twisted flux tube—toroidal field outside this region would ‘unwind’ through the atmosphere and disappear.

Qualitatively the difference in equilibria resulting from differing radial energy distributions in the initial conditions can be understood in the following way. If the energy distribution is flatter, the axis of the circular flux tube in an axisymmetric equilibrium will be closer to the surface of the star and the bulk of the poloidal flux goes through the surface, leaving a smaller volume in which the toroidal component can reside. Beyond some threshold, this means that the toroidal field is not able to fulfil its role in stabilizing the instability seen in purely poloidal fields, and the field buckles, the flux tube first attaining a shape reminiscent of the seam on a tennis ball and then something more complex. This lengthening of the flux tube (at constant volume) increases the toroidal (axial) field strength and decreases the poloidal field strength, until stability is regained. The same process can also be thought of in terms of the tension in a flux tube, which is equal to $T=(2B_{\mathrm{ax}}^{2}-B_{h}^{2})a^{2}/8$ where *a* is the radius of the tube, and *B*_ax_ and *B*_h_ are the RMS axial (toroidal) and hoop (poloidal) components of the field.^5^ In the simple axisymmetric equilibrium, the tension in the tube must be positive. If the tube is too close to the surface, there is not enough space for the toroidal field and the tension can become negative, causing the tube to buckle into a more complex shape until the lengthening of the tube causes the tension to go to zero. A fuller discussion is given in Braithwaite [93].

**Figure 13.**
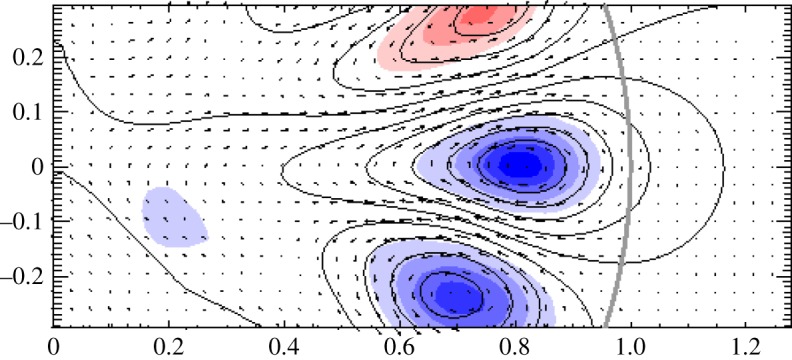
Cross-section of a non-axisymmetric equilibrium. The curved grey line towards the right is the surface of the star and the centre of the star is on the left; the coordinate system used for the plot is cylindrical. The blue/red shading represents the toroidal field component (out of/into the page) multiplied by the cylindrical radius. The poloidal component (in the plane of the page) is represented by the arrows and by contours of its scalar potential, calculated by ignoring spatial derivatives perpendicular to the plane. In fact, it can be seen that the arrows are very nearly parallel to the contours of the scalar potential, showing that the length scale of variation in the direction perpendicular to the page is much greater than the length scale in the plane of the page, i.e. that the flux tubes meander around the star over scales much greater than their width. Note also that neighbouring flux tubes can have toroidal field in either the same or opposite directions: as the toroidal field is absent from the space between the tubes, one tube is not ‘aware’ of the direction of toroidal field in its neighbours, so the equilibrium and stability properties are independent of its direction. Figure from Braithwaite [93].

Figure 13 shows a cross-section of a non-axisymmetric equilibrium found in a simulation. Note how in this figure, as well as in the cross-sections of axisymmetric equilibria in figure 11, we can see that the poloidal field is parallel to contours of the toroidal field multiplied by cylindrical radius. This condition was derived analytically in the case of axisymmetric equilibria from the need for the toroidal part of the Lorentz force to vanish [94,95], and the same condition applies in non-axisymmetric equilibria.

### 3.6. Recent analytic work

There has recently been renewed interest in finding stable equilibria with analytic and semi-analytic methods. In stably stratified stars, the range of possible equilibria is large or perhaps even essentially unlimited within the zero-divergence constraint (as described above in §3.2), although of course only some of the subset will be stable. To find analytic equilibria, various assumptions and choices are made to constrain the solutions. For instance, all analytic works so far have assumed axisymmetry.

Furthermore, except for a few very recent papers (see below), all works have assumed a barotropic equation of state (e.g. [96–99]), which represents a significant restriction on the range of equilibria, as described in §3.2. The barotropic assumption is also quite artificial: regions in stars are either stably stratified or convective. In a convective region in a non-rotating star the stratification is nearly barotropic, but convective flows are incompatible with static field configurations. In a rotating star, convective zones are not even nearly barotropic and thermal winds arise. The parameter space in between convection and stable stratification is a ‘set of measure zero’, of rather academic interest. Pursuing the line of thought nevertheless, taking the momentum equation (3.2), setting the left-hand side to zero (as in equilibrium) and taking the curl, gives

3.3 $$\text{0}=\nabla \times \left(-\frac{1}{\rho }\nabla P\right)+\nabla \times \text{g}+\nabla \times \left(\frac{1}{4\pi \rho }(\nabla \times \text{B})\times \text{B}\right).$$

Gravity being a conservative force, the curl of **g** vanishes. With a barotropic equation of state *ρ*=*ρ*(*P*), the pressure gradient force −(1/*ρ*)**∇***P*=−**∇***h*, where *h*=*h*(*P*) is a new variable; the curl of this force is then obviously zero. We are left with a condition of curl-free Lorentz acceleration

3.4 $$\nabla \times \left[(\nabla \times \text{B})\times \frac{\text{B}}{\rho }\right]=\text{0}.$$

In other words, the Lorentz acceleration must have vanishing curl because it is balanced by two other forces with vanishing curl. Thus, the barotropic equation of state imposes a restriction on the equilibrium which does not exist with an equation of state *ρ*=*ρ*(*P*,*T*). Assuming axisymmetry and a barotropic EOS, finding an equilibrium is a matter of solving the Grad–Shafranov equation, derived from the equilibrium condition that comes from setting the l.h.s. of (3.2) to zero.

**Figure 14.**
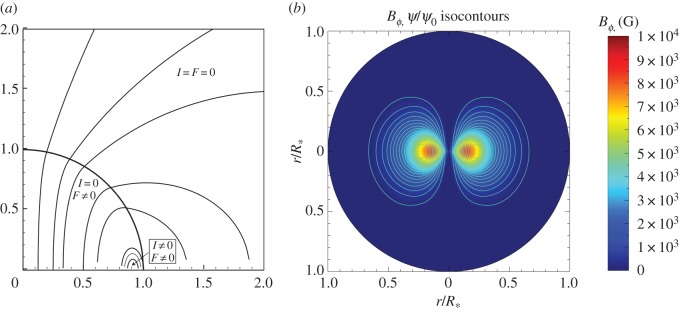
Magnetic equilibria found analytically. (*a*) An equilibrium from Lyutikov [98] where the toroidal field occupies a volume around the neutral line, and (*b*) an equilibrium from Duez *et al.* [100], where the magnetic field is confined entirely to the star. Both studies use a barotropic equation of state and Newtonian gravity.

Most studies have contructed simple twisted-torus equilibria of the form in the upper part of figure 12, with some more complex axisymmetric equilibria with two or more tori. Some examples are shown in figure 14.

A common feature of the simple equilibria is that the volume containing closed poloidal lines and toroidal field is rather small, the neutral line (where the poloidal field vanishes) being close to the stellar surface (e.g. [98]; figure 14). This is possibly something to do with the requirement that the equilibrium can be expressed mathematically as the sum of low-order spherical harmonics—indeed the interior field is often matched to a pure dipole field outside the star. Physically, an equilibrium can form out of an initial field which has most of its flux buried away from the stellar surface and so more deeply buried equilibria must be possible, and such buried equilibria are found in simulations (figure 11). Alternatively, this may have to do with the use of a barotropic EOS.

Other authors (e.g. [100,101]) have found equilibria where the poloidal field does not penetrate through the stellar surface at all, so that toroidal field occupies the entire volume of the star. Strictly speaking, these are of course of academic interest if the application is to objects with an observed field at the surface, but equilibria in nature may have only a modest fraction of their flux passing through the surface, if the magnetic energy is relatively concentrated in the middle of the star.

Several models ([102–104] inter alia) include a current sheet at the stellar surface. This means that the Lorentz force is infinite, which is impossible in nature, especially at a location where the fluid density goes to zero. In, for instance, Broderick & Narayan [102] the current sheet is an unavoidable consequence of the assumption made that the field in the interior is force-free (i.e. **j**×**B**=**0**). To see this, recall a classical result: the ‘vanishing force-free field theorem’. It says that *a magnetic field which is force-free everywhere in space vanishes identically*. Force-free fields can exist only by virtue of a surface where the Maxwell stress in the field is taken up. (For the three-line proof see [1], also reproduced in [2].) The Lorentz force density (the divergence of this stress) may vanish; the stress itself, however, vanishes only when the field itself vanishes.

While most studies use the approximations that (i) the field is too weak to have a significant effect on the shape of the star, (ii) the star is not rotationally flattened and (iii) general relativistic effects can be ignored, some authors drop these assumptions. For instance, Fujisawa *et al.* [99] have a rotating model with a strong magnetic field and Ciolfi *et al.* [97,105] include general relativity (relevant in the context of neutron stars). Perhaps reassuringly, including these effects does not seem to result in any qualitative difference to the geometry of the equilibria found. Also interesting in the context of neutron-star magnetic equilibria is the Hall effect, which is essentially an extra term in the induction equation (3.1) to account for the velocity difference between the electron fluid, to which the magnetic field is frozen, and the bulk flow (e.g. Gourgouliatos *et al.* [106]).

#### 3.6.1. Stability

Having found an equilibrium, one needs to check its stability. Some authors have tried to ensure the stability of their equilibria by finding an energy minimum with respect to some invariants. A popular invariant to use is magnetic helicity H=∫A⋅B dV, where **A** is the vector potential defined by **∇**×**A**=**B**, which is approximately conserved in a highly conducting fluid. For instance, Ciolfi *et al.* [105] use a maximum helicity argument to find the ratio between poloidal and toroidal field strengths. This approach was also used by, for instance, Broderick & Narayan [102] and Duez & Mathis [107], who also introduce further higher-order invariants. The efficacy of these higher-order invariants is not completely established; indeed, it is not even certain to what degree helicity should be conserved when a significant fraction of the flux passes through the stellar surface, above which helicity conservation breaks down.

As mentioned above, almost all of the analytic work has assumed a barotropic equation of state, such as would apply to a convective star. This is done mainly for mathematical convenience; however, it may be of physical relevance in neutron stars, where beta processes eliminate the stable stratification over some timescale [108]. In a barotropic star, one can imagine that an equilibrium magnetic field might be able to ‘hold itself down’ against buoyancy somehow by means of magnetic tension. This would presumably only work if the magnetic field has organized itself globally, with buoyancy acting in opposite directions on opposite sides of the star. Physically relevant of course is not just the question of whether equilibria are possible in principle, but whether they can actually form from realistic initial conditions. It was suggested by Braithwaite [109] that buoyancy, acting on a disorganized initial magnetic field, pushes the magnetic field to the surface faster than it is able to organize itself into an equilibrium. Using star-in-box numerical methods Mitchell *et al.* [110] have also investigated this issue. They use an ideal gas EOS with a heating/cooling term which maintains a uniform entropy in the star’s interior. This removes the stabilizing effect of the stratification and so is equivalent to a barotropic equation of state, but is numerically easier to implement. It is found that the small-scale random magnetic field which does evolve into a stable equilibrium in a stably stratified star does *not* reach an equilibrium in an isentropic star.

It seems possible in principle, however, that an equilibrium could form while the neutron star is non-barotropic, and could somehow adjust to the changing structure of the star, remaining in a quasi-statically evolving stable equilibrium as the star becomes barotropic. Mitchell *et al.* [111] construct various mixed toroidal-poloidal axisymmetric torus fields in a barotropic star, and use numerical methods to test their stability in the linear regime. All of the equilibria constructed prove to be unstable, and the authors tentatively suggest that stable equilibria might not exist in barotropic stars. The decay involves global-scale modes and happens on an Alfvén timescale. The condition for instability is simply that the buoyancy frequency is less than the inverse Alfvén timescale. In the light of these results, it is probably safe to assume that barotropic stars cannot host MHD equilibria. As neutron stars do host magnetic fields, it seems they cannot be perfectly barotropic, or that the crust plays an important role.

### 3.7. The ‘failed fossil’ hypothesis

Above, it was described how an arbitrary magnetic field evolves towards an equilibrium on an Alfvén timescale *τ*_A_, but the discussion ignored any (solid-body) rotation of the star, which adds a Coriolis force −2***Ω***×**u** to the momentum equation (3.2). As a general principle in MHD, it can be shown [112] that if the rotation is slow, such that *Ωτ*_A_≪1, the rotation has little effect on the evolution and stability of magnetic fields; this can also be seen from a comparison of the relative sizes of terms in the momentum equation. If however *Ωτ*_A_≫1, then one expects the Lorentz force to be balanced not by inertia but by the Coriolis force, and a comparison of the sizes of these two terms shows that the evolution timescale is no longer equal to the Alfvén timescale *τ*_evol_∼*τ*_A_ but instead $\tau_{\mathrm{evol}}∼\tau_{A}^{2}\Omega$. This general principle is seen in various contexts in MHD; an early reference is Chandrasekhar [113], sections 84 and after, and fig. 101 and it is seen for instance in the growth rates of various instabilities such as the Tayler instability [86,87]. The idea of the Coriolis force, rather than inertia, balancing whatever is driving fluid motion, is of course also well known from atmospheric physics (quasi-geostrophic balance, e.g. [114]).

As described by Braithwaite & Cantiello [115], this principle should also apply to the relaxation of an arbitrary initial magnetic field in a star towards an equilibrium. In a non-rotating star the field evolves on a timescale *τ*_A_, its energy falling as it does so. If its energy falls by a large factor as it does so (as seems likely; see discussion in §4) then *τ*_A_ will increase by a large factor as the relaxation progresses, and the time taken to reach equilibrium can be approximated simply to the Alfvén timescale at equilibrium. In a rotating star the evolution timescale becomes $\tau_{A}^{2}\Omega$, and increases in the same way as relaxation progresses. Putting in some numbers, it takes 1 year to form an equilibrium of strength 10 kG in a non-rotating star, but 5×10^11^ yr to form an equilibrium of 1 G in a star rotating with a period of 12 h. It may be that in stars lacking strong fields, the magnetic fields are still evolving ‘dynamically’ towards a weak equilibrium. During this time, the evolution timescale *τ*^2^_A_*Ω* will be roughly equal to the age of the star, and so given the age and rotation period of a star, it should be possible to calculate the field strength. With Vega, assuming a rotation period of 12 h and an age of 4×10^8^ years, this argument predicts a field strength of 20 G; for Sirius it predicts about 7 G. It may well be then that Vega and Sirius (see §2.2.1) contain a non-equilibrium field undergoing dynamic evolution. It is easy to reconcile the predictions with observed field strengths of 0.6 and 0.2 G: the observations will underestimate the strength of a smaller-scale field, and one would naturally expect the field strength on the surface to be lower than the volume-average predicted by the theory.

Finally, if it can be assumed in all stars which hosted a pre-main-sequence convective dynamo, that the dynamo leaves behind a magnetic field, then a magnetic field of this order of magnitude should be visible at the surface of all such stars during the main sequence. However, this assumption is not certain—the slow retreat of the pre-main-sequence convective envelope containing a time-dependent dynamo will leave a field of small radial length scale, causing the field to decay more quickly via magnetic diffusion. Its survival will also be affected by processes like meridional circulation and differential rotation. See §3.8 for a more detailed discussion of this point.

### 3.8. The evolution of fossil fields

As mentioned in §§2.1.4 and 2.2.2, the strength of the fields observed in magnetic early-type stars falls during the main sequence, and it falls faster than expected from simple flux conservation while the star’s radius increases. Various explanations spring to mind, the most obvious of which is Ohmic diffusion. The global timescale for Ohmic diffusion is of the order of 10^10^ years, somewhat longer than the main-sequence lifetime of the least massive stars in question. It may, however, be possible to massage this timescale downwards, perhaps by making use of the lower conductivity near the surface of the star; conductivity goes as *T*^3/2^. In the absence of other effects, one would expect the electric current associated with the magnetic field to die away reasonably quickly in the outer part of the star, so that after some time the field at the surface is simply a potential-field extrapolation of the field further inside. Depending on the initial geometry and radial distribution of the magnetic field, this could cause the surface field either to rise or fall during the main sequence. This will depend on the origin of the magnetic field. The main weakness of finite conductivity in explaining this decay though is that it is also observed in massive stars with much shorter main-sequence lifetimes and somewhat longer Ohmic timescales than intermediate-mass stars.

Another possibility is the combination of buoyancy and thermal diffusion. In short, a magnetic feature is in pressure balance with its less-strongly magnetized surroundings, and so its gas pressure must be lower; to avoid moving on a dynamic timescale its temperature must therefore be lower than its surroundings. Heat consequently diffuses into it, resulting in a buoyant rise to the surface [65,67,85,93,116]. Note that this mechanism is distinct from the so-called buoyancy instability (or Parker instability) where diffusion is not required. In the low-density environment near the surface of a star where heat diffusion is very efficient, the rise is limited by aerodynamic drag and takes place at the Alfvén speed (§6.5). Deeper down, the process is limited by heat diffusion, and for a global magnetic field structure its timescale can be expressed in terms of the Kelvin–Helmholtz timescale and the plasma-*β* as *τ*_decay_∼*τ*_KH_*β* [93]. On the one hand, this would immediately explain why a similar flux decay is seen in A, B and O stars, as the thermal timescale is roughly the same fraction of the main-sequence lifetime and *β* falls in roughly the same range at all spectral types. On the other hand, it might be tricky to get this process to work fast enough: even assuming that the interior field is 10 times stronger than the surface field, the timescale for the most strongly magnetized stars (e.g. an Ap star with a 30 kG field) would be comparable with the main-sequence lifetime. As the timescale goes as *B*^−2^, the effect in stars with much weaker fields would be negligible. However, as with Ohmic diffusion, it may be possible to massage the numbers in the light of the fact that the thermal diffusion timescale is much shorter near the surface of the star.

Another possibility is meridional circulation. Its characteristic timescale is the Eddington–Sweet time, *τ*_decay_∼*τ*_KH_*E*_grav_/*E*_rot_, making it occupy roughly the same range, as a fraction of main-sequence lifetime, at all masses. The geometry of this flow has been clarified in the seminal work of Zahn [117]. Note the similarity with the timescale of diffusive buoyancy in the previous paragraph—the only difference is that rotational energy has replaced magnetic energy. As the ratio of energies in this case can be much closer to unity than in the previous case, the timescale can also be much smaller, indeed the fast-rotating magnetic stars should experience relatively fast decay, unless the magnetic field finds some way to coexist alongside the meridional flow. It may be, though, that this flow is simply inhibited by the magnetic stress in the fossil field. Essentially nothing is present in the literature regarding how this inhibition might work. A first guess might be that the magnetic energy would have to be greater than the circulation kinetic energy, leading to the stability condition $E_{\mathrm{mag}}>MR^{2}\tau_{\mathrm{KH}}^{-2}E_{\mathrm{grav}}^{-2}E_{\mathrm{rot}}^{2}$, or

3.5 $$\beta <\frac{\tau_{\mathrm{KH}}^{2}}{\tau_{\mathrm{rot}}^{2}}\frac{E_{\mathrm{grav}}^{3}}{E_{\mathrm{rot}}^{3}}.$$

Alternatively, an interaction with the convective core could be the crucial process—the convective motion and waves that it sends into the radiative envelope could somehow result in an enhanced diffusion. In this case, one would certainly expect a correlation with mass, as the core is very small in late A stars but reaches to around a third of the stellar radius in O stars. This topic is explored in §6.3. In any case, important clues will come if/when correlations are observed between flux decay and mass, rotation and field strength.

## 4. The origin of fossil fields

The discussion in the previous section begs a question: where does the variation in magnetic fields in otherwise similar stars come from? An important clue must be the observed extreme range of field strengths in A stars, with roughly ‘equal numbers per decade’ between 200 and 2×10^4^ G, as well as the bimodality, with no stars having fields between a few gauss and 200 G [11]. The existence of very weak fields such as in Vega and Sirius (§2.2.1) highlights the suspicion that the field in the protostellar cloud from which a star forms is not particularly relevant in determining the fields observed in stars (contrary to the classical view of the origin of fossil fields). There are various ways in which one might explain the observed range in field strength; the relevant processes are discussed below approximately in order of decreasing scale and/or increasing time.

### 4.1. Variations within the interstellar medium

According to the traditional model, variations in magnetic field strength in the interstellar medium (ISM) are simply carried forwards into the star. In the light of recent results though, including the very weak fields in Vega and Sirius, this scenario, at least in its simplest form, now looks very unlikely—the range in field strengths in stars is far greater than that in the ISM. In addition, this model requires an additional ingredient to produce the observed bimodality between Ap and other A stars. It is perhaps a clue that the lower threshold of 200 G in Ap stars is the same as the equipartition field strength at the photosphere—in the merger hypothesis (§4.6); this might have to be a coincidence. Also, this model is compatible with the lack of magnetic stars observed in binaries, because collapsing cloud cores with a strong magnetic field will spin down efficiently, whereas cores lacking a strong field will retain too much angular momentum to form a single star and so form a binary. This effect has been seen in simulations [118]. To summarize, the simple ISM-variation model ignores much of the star formation process and so, alone, it will not explain what we see in stars. It may play some role however; in any case it is worthwhile to take a more detailed look at star formation from the perspective of magnetic field evolution.

### 4.2. Core collapse

In star formation, the relative strength of the gravitational and magnetic fields is often expressed as a dimensionless mass-to-flux ratio, defined as λ≡2*πG*^1/2^*M*/*Φ*, where *M* and *Φ* are the mass and magnetic flux, or locally in a disc context as 2*πG*^1/2^*Σ*/*B*_*z*_ where *B*_*z*_ and *Σ* are the field normal to the disc, and surface density; this ratio is conserved if flux freezing is valid. It is related to the gravitational and magnetic energies by (ignoring factors of the order of unity) λ^2^=|*E*_grav_|/*E*_mag_. A cloud with λ≳1 is said to be ‘magnetically supercritical’ and will collapse, in the absence of significant thermal or rotational energy. Conversely, a cloud with λ≲1 is ‘magnetically subcritical’ and the magnetic field supports the cloud against gravity. Once a cloud has become supercritical, it can collapse dynamically. Magnetic braking becomes ineffective once the collapse is super-Alfvénic, so the rotational energy becomes larger in relation to the other energies. Normally, this leads to formation of a disc of radius 100–1000 arb. units.

### 4.3. The role of the accretion disc

There is strong evidence that discs contain strong, ordered, net poloidal flux—there are direct measurements of the magnetic field in protostellar discs at various radii from 1000 arb. units right down to 0.05 arb. units [119–121] and the mass-to-flux ratio is always found to be of the order of unity.^6^

There is additional evidence for the presence of ordered magnetic fields in discs. Systems ranging from protostars to active galactic nuclei usually (not always, and not all of them) show evidence of a fast outflow in the form of a collimated jet. The default model for its origin is the rotation of an ordered magnetic field in the inner regions of the disc. That is, a field crossing the disc with a uniform polarity over a significant region around the central object. Models assuming the existence of such an ordered field (as opposed to the small-scale field of mixed polarities generated in magnetorotational instability (MRI) turbulence) have been particularly successful in producing fast magnetically driven outflows. Accepting this as evidence for the existence of such ordered fields, they might also be the fields that are accreted to form magnetic A, B and O stars.

The origin of the ordered field in discs is less certain. An important constraint on the possibilities is the fact that the net magnetic flux crossing an accretion disc is a conserved quantity (a direct consequence of div **B**=0). It can change only by field lines entering or leaving the disc through its outer boundary, i.e. any net flux in a disc must be accreted from the ISM.

Given the strong ordered fields in discs, it is something of a puzzle that in stars we observe mass-to-flux ratios λ∼10^3^ in the most strongly magnetized Ap stars and ratios up to at least 10^8^ in other stars. In other words, we have an extra phenomenon to explain: why even the most strongly magnetized stars have such weak fields. If in the steady state, the star is accreting mass and flux in the ratio λ_*_, then mass and flux must be passing through each surface of constant radius in the disc in the same ratio (ignoring outflow), even though the local ratio will in general be much lower λ(*r*)≪λ_*_, requiring almost perfect slippage at all radii. Either there is a fundamental problem accreting flux through a disc, or there is a bottleneck further in through which magnetic flux cannot be accreted, located either in the star’s magnetosphere or in the star itself.

This may be related somehow to the fact that accretion discs are turbulent. Simple estimates show that accretion of an external field is very inefficient if the disc has a magnetic diffusivity similar to the turbulent viscosity that enables the accretion [122]. Numerical simulations [123] show that this is in fact a good approximation for magnetorotational turbulence. Though intuitively appealing, accretion of the field of a protostellar cloud as the source of Ap star fields is therefore not an obvious possibility.

The flux bundles that drive jets from the inner regions of the disc, inferred indirectly from observations, must somehow be due to a more subtle process. Numerical simulations for the case of accretion onto black holes (cf. [124], and references therein) have shown that ‘a flux bundle’ of uniform polarity in the inner disc can persist against outward diffusion in a turbulent disc. These results are still somewhat artificial as the flux of the bundle in such simulations depends on the magnetic field assumed in the initial conditions. The speculation is (cf. [125]) that this flux itself starts as a random magnetic fluctuation further out in the disc, which somehow is advected inward with the accretion flow (e.g. [126]).

It may be then that magnetic stars have accreted random magnetic features from the accretion disc. Whether such a scenario is realistic is an open question (for recent suggestions in this direction see [127]). If something like this happens, it might explain the apparently unsystematic presence of jets from accretion discs. In the context of A stars, the assumed randomness of the sources in the disc on a range of length and time scales, might perhaps explain the range in field strength of magnetic Ap stars.

### 4.4. Destroying flux in or near the star

Given the evidence for strong ordered fields in the inner part of the accretion disc, it might be necessary to prevent the bulk of it from entering the star. Star–disc interaction seems to be intrinsically complex and is poorly understood; and it may be difficult to accrete flux from the inner edge of the disc. What flux does reach the star, however, is certainly not obliged to remain there in its entirety—excess flux can easily be destroyed after it reaches the star [109]. If the protostar is convective, magnetic field is prevented by its own buoyancy from penetrating into the star, and if the star is already radiative then the quantity of magnetic helicity is crucial.

Magnetic helicity is a global scalar quantity defined as H=∫A⋅B dV, where **A** is the vector potential defined by **∇**×**A**=**B**. It can be shown [128] that this quantity is conserved in the case of infinite conductivity. In plasmas of finite but high conductivity, it has been demonstrated that it is *approximately* conserved, for instance in the laboratory [129,130] and the solar corona [131].

If the magnetic field in a radiative star is allowed to relax, it will evolve into an equilibrium (§3). Once an equilibrium has been reached, energy and helicity are related by *H*=*EL*, where *L* is some length scale which is comparable with the size of the system or, in this context, to the size of the star. Therefore, one can predict the magnetic energy of the equilibrium from the helicity present initially—helicity is a more relevant quantity than initial magnetic energy. The observations then imply that there is an enormous range in the magnetic helicity which stars contain at birth, as well as a possible bimodality.

In the symmetrical ‘hourglass model’ of star formation, the helicity is zero. Any field accumulated from such an hourglass should therefore decay to zero energy (cf. Flowers & Rudermann instability, §3 and figure 8). In reality of course, one expects some asymmetry. However, an unrealistically high degree of symmetry would be required to be left with a field of only 1 G from an initial mass-to-flux ratio of the order of unity.

### 4.5. Pre-main-sequence convection

As they descend from the birth line down the Hayashi track, intermediate-mass stars are fully convective, before turning onto the Henyey track (leftwards on the HR diagram), during which time the convective zone retreats outwards and disappears (e.g. [132]). The situation with stars above very approximately 6*M*_⊙_ is less well understood; they may have a radiative core throughout the pre-main-sequence. In any case, as suggested by Braithwaite [109] and shown more thoroughly by Mitchell *et al.* [111] a star with a constant entropy—as in a convective star—cannot hold onto any previous magnetic field: it rises buoyantly towards and through the surface on a Alfvén timescale. This process does not actually require the convective motion itself, just the flat entropy profile it creates. So, a pre-existing field should be replaced by a convective dynamo field.

As the convective zone retreats towards the surface, it should leave something behind in the radiative core. Assuming that the dynamo fluctuates, as in the case of the Earth and the Sun, layers of alternating polarity are deposited into the growing radiative core, rather like we see in the rocks of the mid-Atlantic ridge, which keep a memory of the polarity of the Earth’s magnetic field at the time when it solidified from magma. However, given that the star is fluid, and evolves on a thermal timescale which is very much greater than the dynamo fluctuation timescale, the layers will be very thin and should annihilate each other almost immediately. It is therefore not clear whether anything could be left behind at all; the best one might hope for is some net north–south asymmetry from $\sqrt{N}$ statistics. One way out of this may be if the convective region retreats more rapidly. This could happen in the inner part of the star: the Schwarzschild criterion is breached first at some intermediate radius and a thin radiative layer forms, cutting off the supply to the interior of deuterium from the ongoing accretion, and resulting in a relatively fast transition to a radiative core [133]. This would not work though in the more massive stars, in which deuterium burning is less important to the stellar structure.

The most obvious serious weakness of pre-main-sequence convection as the origin of Ap-star magnetic fields is that one would expect it to produce a range of magnetic field strengths according to the rotation rate of the star; it is difficult to produce any bimodality. Bimodality could though perhaps be produced afterwards, if some mechanism existed which destroys magnetic fields below some threshold. This highlights the problem that it is not enough to have a theory, whatever it is, that explains a certain typical field strength. The enormous scatter in the observed field values is the actual challenge to theory here.

### 4.6. Mergers

Mergers are a strong candidate to create strong fossil fields in a subset of stars (e.g. [134–136]). Bogomazov & Tutukov [137] suggest that Ap stars may be the result of mergers between binary stars with convective envelopes whose orbits shrink as a result of magnetic braking. This seems more likely on the pre-main-sequence. Ferrario *et al.* [138] point out that the observed correlation between mass and magnetic fraction [12] could be explained by the need for the merger product to be radiative, i.e. on the Henyey track. Exactly how a merger should produce a fossil field is not understood, but we can at least expect plenty of free energy in the form of differential rotation. This model would also explain the lack of close binaries containing an Ap star, although there are one or two peculiar counter-examples to this observational result, for instance the binary HD 200405 with a period of 1.6 days. Even periods of 3 days would be tricky to explain as the result of a merger in a triple system; it might be necessary to reduce the orbital period of the resulting binary after the merger. This might be related to a similar issue for the merger hypothesis, namely that a merger product will initially be rotating close to break-up but that magnetic stars are observed to rotate slowly. In both cases, angular momentum must be extracted. One could imagine perhaps that the material ejected in a merger, which is estimated to be around 10% of the total mass of the two merging stars, could absorb angular momentum as it flows outwards. Some form of magnetic coupling between the stars and the circumstellar material might be important, just as is thought to work to slow down the rotation of single magnetic stars, as discussed in the next section.

### 4.7. The rotation periods

Apart from the large range in field strengths in radiative stars, we also want to explain the range in rotation periods. On the one hand, we need an explanation of how sufficient angular momentum can be removed to form a star at all, but on the other hand, we need to form some stars rotating at close to break-up and others with periods of several decades or more. The correlation between the presence of a significant magnetic field and rotation period is strong, but there is still a large range in rotation period among stars with similar field strengths. How is it possible at all to spin a star down to a rotation period of 50 years, or in other words up to a Keplerian co-rotation radius of ≈17 AU (assuming *M*=2*M*_⊙_)? Assuming a stellar radius *R*=2*R*_⊙_, a surface dipole of 3 kG and the standard *r*^−3^ radial dependence, we arrive at just 0.5 μG at the co-rotation radius—it is obviously a major challenge for any kind of disc-locking model, where the co-rotation and magnetospheric (Alfvén) radii are comparable, for a magnetic field weaker than the galactic average to be in equipartition with gas of much greater density than the galactic average.

## 5. Magnetic fields and differential rotation

As strong stable fields are already found among pre-MS (Herbig Ae-Be) stars, their origin must lie in earlier phases of star formation, when the protostar was in a state of rapid, possibly differential, rotation. The sequence of events that led to the formation of a stable magnetic field is not known, but may have involved processes of interaction between magnetic field and differential rotation. This interaction takes different paths depending on the relative strength of initial field and initial rotation. As we do not know the star formation process well enough, we have to consider different possibilities.

Taken as a measure of the strength of the magnetic field the Alfvén frequency $\omega_{A}={\bar{v}}_{A}/R$, with ${\bar{v}}_{A}=B/{\left(4\pi \bar{\rho }\right)}^{1/2}$, where *B* is a representative field strength, $\bar{\rho }$ the star’s average density and *R* its radius. If *ω*_A_ is large compared with (a representative value of) the differential rotation rate Δ*Ω*, the evolution of the field configuration under the Lorentz forces is fast and the field will relax to a stable equilibrium, if it exists. On top of this, there will be oscillations with frequencies of the order of *ω*_A_, reflecting the aftermath of the relaxation process and the differential rotation that was present initially.

### 5.1. Phase mixing

These oscillations are then damped by a process of *phase mixing*. As a model for this damping consider an idealized case, where the field is in a stable equilibrium to which an azimuthal flow in the form of differential rotation has been added as an initial condition. The deformation of the field lines in this flow reacts back on the flow; the result is an Alfvénic oscillation. The oscillation period is given by an Alfvén travel time. As the energy of Alfvén modes travels along field lines, neighbouring magnetic surfaces oscillate independently of each other. Their frequencies are in general different, with the result that neighbouring surfaces get out of phase. The length scale in the flow in the direction perpendicular to the surfaces decreases linearly with time. The result is damping of the oscillation on a short timescale. For more detailed discussions of this process, see e.g. Heyvaerts & Priest [139] and the references in Spruit [76].

### 5.2. Rotational expulsion

In the opposite case *ω*_A_≪Δ*Ω*, the differential rotation flow is initially unaffected by the field. The non-axisymmetric component of the field (with respect to the axis of rotation) gets ‘wrapped up’, such that lines of opposite direction get increasingly close together, increasing the rate of magnetic diffusion. As a result magnetic diffusion cancels opposite directions in a finite time. The non-axisymmetric component decays, it is effectively expelled from the region of differential rotation. The process (‘rotational expulsion’, [140]) is similar to the evolution of a weak field in a steady convective cell (‘convective expulsion’, see §6.1.1 below).

This process, if allowed to proceed to completion, will therefore ‘axisymmetrize’ the initial field configuration. In this idealized form, it is probably somewhat academic, however, as the wrapping process increases the field strength linearly with time (the non-axisymmetric as well as the axisymmetric component). It may well happen that magnetic forces become important before magnetic diffusion has become effective (for discussion, see [76]). Magnetic instabilities, Tayler instability being the first to set in, then take over and determine the further evolution (§5.4). As discussed below, this can have important consequences for the rotational properties of the star.

### 5.3. Angular momentum transport in radiative zones

In A stars, the detection limit for large-scale magnetic fields is of the order of a few gauss, and somewhat lower in very bright stars like Vega and Sirius where sub-gauss fields have been found (§2.2.1 above). It is reasonable to assume that the internal field strengths of these stars are rather higher than the measured value at the surface, as stronger smaller-scale fields at the surface would escape detection and as it would not be surprising if the magnetic field were weaker at the surface than deeper inside the star; the internal field strength even in these ‘non-magnetic’ stars is likely to be of the order of 10 G or perhaps more (see also §3.7).

Even fields below current detection limits for these stars can have dramatic effects though on the internal rotation of stars. If *r* is the distance from the centre of the star, the torque exerted by Maxwell stresses in a field with toroidal (azimuthal) component *B*_*ϕ*_ and radial component *B*_r_ is of the order of *r*^3^*B*_*ϕ*_*B*_r_. The torque in a ‘geometric mean’ field $\bar{B}={\left\langle B_{\phi }B_{r}\right\rangle }^{1/2}$ of the order of 1 G is sufficient to redistribute angular momentum on a timescale of the star’s main-sequence lifetime, and to keep the core co-rotating with its envelope as the star spins down by a stellar wind torque (a classical argument dating from the 1950s).

This idea may, however, be a little over-simplistic. As mentioned above, the MHD instabilities are expected to set in. This could result in a magnetic dynamo.

### 5.4. Dynamos in radiative zones

Fields generated by some form of dynamo activity have traditionally been associated with convective envelopes, to the extent that dynamo action in stars was considered equivalent with a process of interaction between magnetic fields, convective flows and differential rotation. This is only one of the possibilities, however. In fact, differential rotation alone is sufficient to produce magnetic fields. The most well known example is that of magnetorotational fields generated in accretion discs [141]. The idealization of an accretion disc in this case is a laminar shear in a rotating flow, with a rotation rate *Ω* decreasing with distance *r*. Dynamo action triggered by MRI quickly (10–20 rotation periods) generates a fluctuating field with a small-scale radial length scale (*l*∼*c*_s_/*Ω*, where *c*_s_ is the sound speed, comparable with the thickness of the accretion disc).

Given a sufficiently high magnetic Reynolds number, the energy source of differential rotation is sufficient for field generation, in the presence of a dynamical instability of the magnetic field itself. In discs, the magnetic instabilities involved in ‘closing the dynamo cycle’ are magnetorotational instability [141] and magnetic buoyancy instability [142,143]. In the case of the solar cycle, the phenomenology strongly indicates magnetic buoyancy as the main ingredient in closing the dynamo cycle (see review in [144]). This contrasts with conventional turbulent mean-field views of the solar cycle (e.g. [145]); for a critical discussion see Spruit [146,147].

In the radiative interior of a star, the high stability of the stratification allows buoyant instability only at very high field strengths (cf. the review in [76]). Instead, in such a stable stratification a pinch-type instability is likely to be the first to set in. A dynamo cycle operating on differential rotation combined with this ‘Tayler instability’ has been described in Spruit [148]. Its application to the solar interior predicts field generation at a level just enough to exert the torques needed to keep the core in nearly uniform rotation and to transport the angular momentum extracted by the solar wind [148,149]. The magnetic field generated in this model is extremely anisotropic: in the radial (*r*) direction, the length scale for changes of sign of field line direction is very small. It should thus be regarded as a ’small-scale dynamo’ instead of a global one.^7^ This reflects the dominant role of the stable stratification, but also the nature of Tayler instability, which is a local one in the *r* and *θ* directions. In the azimuthal direction, however, its length scale is large, dominated by the fastest growing non-axisymmetric Tayler mode, *m*=1.

Tests of this dynamo cycle through some form of numerical simulation would be desirable. In a proof-of-principle simulation by Braithwaite [150], a dynamo cycle in a differentially rotating stable stratification including thermal diffusion was observed with properties as predicted. Simulations for realistic stellar conditions present serious obstacles, however. The predicted fields are very weak compared with the stability of the stratification, while the cycle time is very large and its length scale small compared with the other physical scales of the problem. A widely cited simulation by Zahn *et al.* [151], claimed to be valid for the physical conditions in the Sun, did not yield dynamo action. Inspection of the parameter values actually used in this simulation shows that the negative result is caused by damping of magnetic perturbations by the high magnetic diffusivity assumed (orders of magnitude off). With the thermal and magnetic diffusivities used, the differential rotation in this simulation is actually three orders of magnitude below the threshold for dynamo action (equation 27 in [148]). Zahn *et al.* [151] is not relevant, neither for questions of existence or otherwise of differential rotation-driven dynamos in stably stratified zones of stars, nor as a test of a possible dynamo cycle.

The large range in length and time scales involved makes simulations for realistic conditions in stellar interiors impossible to achieve at present. The dynamo mechanism should be directly testable, however, by appropriate simulations which lie in a parameter space that satisfies the minimum criteria (such as those that were reported already in [150]).

Recently, Jouve *et al.* [152] performed simulations of a differentially rotating star with an incompressible constant-density equation of state, finding that the MRI is the dominant dynamo process. In a more realistic stably stratified star, it is not immediately obvious whether the MRI or the Tayler instability should dominate. While under adiabatic conditions the Tayler instability is the first to set in, the situation may be different once thermal diffusion is included.

### 5.5. Observational clues

Only very indirect observational evidence is available for or against the existence of magnetic fields in radiative interiors. The nearly uniform rotation of the solar interior, as well as its co-rotation with the convective envelope, have long posed the strongest constraint on possible angular momentum transport mechanisms.^8^ In addition, the rotation rates of the end products of stellar evolution, the white dwarfs and neutron stars, may provide clues. To the extent that the rotation of these stars descends directly from their progenitors (AGB stars and pre-supernovae), they also contain information regarding the degree to which the cores of the progenitors were coupled to their envelopes. The very high effectiveness of Maxwell stress at transporting momentum makes magnetic fields the natural candidate. Transport of angular momentum by internal gravity waves (e.g. [153]) may also be an important mechanism; however, for recent theoretical developments, see [154].

It is somewhat uncertain, however, whether there is a direct connection between the end products and the internal rotation of their progenitors. The observed asymmetries in planetary nebulae, for example, indicate highly asymmetric mass loss in the final evolution stages of AGB stars. Such asymmetric ‘kicks’ may have reset the angular momentum of the cores, such that the rotation rates of WD actually reflect the ‘kicks’ imparted by the last few mass loss events rather than the initial core rotation [155]. Supernova kicks [156] may also be the main process determining of the rotation of neutron stars [157].

**Figure 15.**
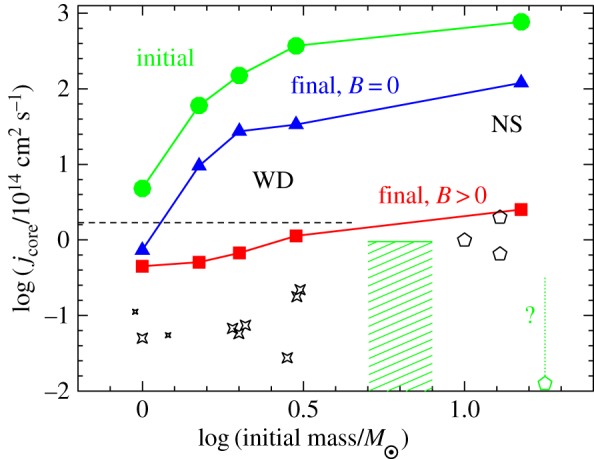
Specific angular momentum of white dwarfs (WD) and neutron stars (NS) as a function of initial mass of the progenitor star, as computed by Suijs *et al.* [158]. Green circles: initial core angular momentum, blue triangles: including known hydrodynamic angular momentum transport mechanisms, red squares: including the Tayler–Spruit magnetic torque prescription. The dashed horizontal line indicates the spectroscopic upper limit on the whitedwarf spins obtained by Berger *et al.* [159]. Star symbols represent astroseismic measurements from ZZ Ceti stars and the green hatched area is populated by magnetic white dwarfs. The three black open pentagons correspond to the youngest Galactic neutron stars, while the green pentagon is thought to roughly correspond to magnetars, where the vertical-dotted green line indicates the possibility that magnetars are born rotating faster. See Suijs *et al.* [158] for more details.

Leaving these complications aside, stellar evolution calculations are used to make predictions of the rotation rates of end products by including (parametrizations of) known mechanisms of angular momentum transport such as meridional circulation (e.g. [117]) and hydrodynamic instabilities. Figure 15 shows results from evolution calculations of massive and intermediate-mass stars. Including the hydrodynamic processes predicts rotation rates that are too high by ∼2 orders of magnitude, clearly indicating that much more effective processes of angular momentum transport must be present in stars. When magnetic torques according to Spruit [148] (see also §5.4) are included, agreement with observations is better, but discrepancy of 1 order of magnitude nevertheless is still present.

#### 5.5.1. Asteroseismic results

**Figure 16.**
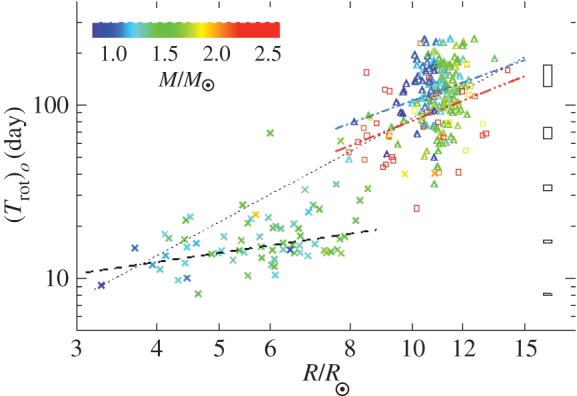
Mean period of core rotation as a function of the asteroseismic stellar radius, in log–log scale. Crosses correspond to RGB stars, triangles to clump stars, and squares to secondary clump stars. The colour code gives the mass estimated from the asteroseismic global parameters. The dotted line indicates a rotation period varying as *R*^2^. The dashed (dot-dashed, triple-dotted-dashed) line indicates the fit of RGB (clump, secondary clump) core rotation period. The rectangles in the right side indicate the typical error boxes, as a function of the rotation period. From Mosser *et al.* [160].

The new asteroseismic results of giants and sub-giants from the Kepler mission have greatly expanded the evidence on the internal rotation of stars other than the Sun [160]. The cores of these stars rotate faster than their envelopes, with typical periods of 10 to 200 days (figure 16). This shows a degree of decoupling between envelope and interior. The torques required to explain these rotation rates are still much stronger than can be explained by the known non-magnetic processes, however, with the possible exception of angular momentum transport by internal gravity waves, cf. [161–164]. Results from stellar evolution calculations using the estimate in Spruit [148] can be compared with the Kepler rate of figure 16. As with the rotation rates of the end products, the predicted rotation rates are up to a factor of 10 too high. (The fact that the disagreement is by a similar factor in both cases may be a coincidence.)

## 6. Interaction between convective and radiative zones

In this section, we explore the interaction of steady magnetic fields with convection. In particular, we look at the possibility of steady fields in the radiative interiors of solar-type stars, shielded from becoming observable at the surface (§6.1) and how these might interact with the convective envelope above (§6.2). In §6.3, we look at at how fossil fields in the radiative envelopes of early-type stars might interact with their convective core; and finally, we take a brief look at fully convective stars and the possibility of producing magnetic fields in subsurface convective layers in early-type stars.

In contrast with A, B and O stars, among which a significant minority has a detectable field and the rest of the population has at most very weak fields, the cooler stars (those with convective envelopes) seem *all* to display magnetic fields, but never of the stable kind seen in A, B and O stars. In addition, there is apparently some difference among the cooler stars, between magnetic fields in stars such as the Sun which have a radiative core and a convective envelope, and in fully convective stars (this is looked at below in §6.4).

For observation results, the reader is referred to recent spectropolarimetric surveys [165,166] which have provided excellent results on magnetic properties of late-type stars. Also Pizzolato *et al.* [167], Wright *et al.* [168] obtained interesting results which link dynamo activity to rotation. In solar-type stars, the magnetic fields vary much like the Sun’s field, without a consistent steady component; the small-scale field is much stronger than the large-scale field. Upper limits on a steady dipole component on the Sun (which would be observable as a North–South asymmetry during the solar cycle) are probably of the order of a few gauss.

In these stars, a field anchored in the radiative core could in principle be present, but if it is, it seems not to manifest itself at the surface. Two kinds of explanation suggest themselves. A convective envelope might somehow be incompatible with the presence of a fossil field, either by preventing it from forming during star formation, or by destroying it soon after (§6.2 below). Alternatively, it might be that a fossil field is actually still present in (some) solar type stars, but somehow confined or ‘shielded’ by the convection zone (§6.1).

### 6.1. Confinement of steady fields in the radiative interior of sun-like stars

To the extent that a steady internal field connects to the convective envelope it might become detectable at the surface as a time independent component, superposed on the cyclic dynamo-generated field characteristic of stars with convective envelopes. The Sun provides some limits on this possibility. Its cyclic dipole component has an amplitude of about 20 G at the magnetic poles [169]. In most cycles, some asymmetries are seen between the North and South Hemispheres, but no signal of a long-term average polarity has been reported. The implied detection limit is probably of the order of a few gauss. This indicates either that a steady field in the solar interior has a dipolar component below a few gauss, or that a (possibly stronger) field is somehow actively shielded by the convection zone.

A steady, shielded field (called ‘inevitable’ by its authors) was discussed by Gough & McIntyre [170]. Their model assumes a meridional circulation near the base of the convection zone, downward at the poles and the equator and upward at mid-latitudes. A magnetic field in the radiative zone, such that the field lines are parallel to its interface with the convective zone, is in contact with this circulation. The authors find a steady solution to a set of reduced equations, with the property that the interior field does not spread into the convection zone.

This is a somewhat surprising result. At its upper boundary, field lines from the interior spread upward into the convection zone by magnetic diffusion. At the poles and equator of the model, the downward advection of field lines by the circulation opposes this spreading. At its mid-latitude, however, advection in the model is upward, into the convection zone, carrying the field lines in the *same* direction as the spreading by diffusion. This does not depend on the particular configuration of the circulation: there is always at least one region where diffusion and advection both act to spread the field into the flow. A circulation therefore cannot prevent a field in the radiative interior from diffusing into the convective envelope. The conflicting result by Gough and McIntyre is in fact a consequence of the reductions made to the induction equation.^9^

The conclusion from the above is that a field in the radiative interior is inevitably connected to some degree with the convection zone by field lines crossing the interface, even if it does not extend far enough into the convective envelope to become visible at the surface. An internal field can be *shielded* from the visible surface in this way, while at the same time remaining *mechanically coupled* to flows in the envelope, the differential rotation for example. The construction in Gough & McIntyre makes the assumption that the interior field is both shielded and decoupled.

Observations of the interior rotation of the Sun through helioseismology show that the radiative interior has approximately the same specific angular momentum as the convective envelope. As the Sun spins down through angular momentum loss in the solar wind, this observation indicates the existence of an efficient coupling between interior and envelope, on an evolutionary timescale. The coupling resulting from magnetic interaction between interior and envelope naturally fits this observation. With magnetic decoupling as assumed in Gough & McIntyre [170], an additional explanation would be required.

Decoupling is a far stronger assumption than shielding. The contrasting conclusions reached in numerical simulations [171,172] can be traced to confusion of these two concepts.

#### 6.1.1. Shielding: convective expulsion

Shielding of a sufficiently weak internal field could be achieved by a process of *convective expulsion* [173,174]. The first numerical study of expulsion, idealized as a steady circulating flow interacting with a magnetic field was by Weiss [175]. It shows how a convective cell can create a field-free region by pushing the field lines passing through it to the sides of the cell. This happens in two stages. First, the field lines in the cell are ‘wrapped around’ by the flow, changing the field into a complex configuration with changes of direction on small scales. This happens on a short timescale, a few turnover times of the cell. In the second stage, magnetic diffusion reconnects field lines of different direction until the wrapped field has disappeared from the cell. In the field which is now concentrated at the boundaries of the cell the flow is suppressed. The process is effective up to a certain maximum field strength roughly given by equipartition between the magnetic and convective energy densities. In more realistic, time-dependent convective flows, the separation between flows and magnetic fields is observed to be stable once established. An example is the highly fragmented magnetic field seen at the solar surface ([176,177] and references therein).

In the case of shielding of a field in the radiative interior of stars with convective envelopes, both convective flows and meridional circulation would contribute to shielding. But, as argued in the above and in Strugarek *et al.* [171], both would also lead to mechanical coupling of the envelope to the interior by the poloidal field component.

#### 6.1.2. Coupling

The above (§6.1.1) shows that by a convective expulsion process the envelope may be able to effectively shield a field in the radiative interior from manifesting itself at the surface. This does not imply, however, that the envelope and interior are also mechanically decoupled. There is always some connection between the two through field lines diffusing from the interior into the envelope (see §6.1).

The simulations by Acevedo-Arreguin *et al.* [172] focus on the shielding aspect. As in Strugarek *et al.* [171], however, their simulations also show a poloidal field connecting the interior to the envelope. The consequence of this connection is that flows in the envelope, whether in the form of convection or differential rotation, keep the field in the interior in a time-dependent state. It excludes the steady internal field assumed by Gough & McIntyre [170].

The time dependences that can be covered in numerical simulations are of the order of tens of rotation periods of the star. This is to be compared with the age of the fossil field (nine orders of magnitude longer) that is supposed to survive in the interior, in the Gough–McIntyre view. A poloidal field component that is inconsequential on numerically accessible timescales can wreak havoc on the internal field on timescales very short compared with the age of the star.

The differential rotation of the envelope, for example, stretches the connecting poloidal field lines into an azimuthal component. Mestel’s well-known estimate ([178], remark on p. 735) shows that even a field as weak as a few microgauss would, on an evolution timescale, be amplified to a strength sufficient to affect the rotation of the interior. Long before this happens, however, the wound-up azimuthal field will develop magnetically driven instabilities. As argued in Spruit [148], this sequence of events is likely to lead to a self-sustained, time-dependent magnetic field independent of the initial field configuration.

### 6.2. Strong magnetic fields below convective envelopes

Discussions of the Gough–McIntyre field implicitly assume a ‘kinematic’ view of magnetic field evolution: the fields considered are sufficiently weak that their Lorentz forces can be ignored to first-order. One may wonder what stars with convective envelopes would look like if they contained magnetic fields as strong as those of Ap stars in their radiative interiors and Lorentz forces cannot be ignored.

The type of behaviour of a magnetic field in a flow (convective or otherwise) depends on its strength relative to the kinetic energy density in the flow. Field lines connecting the interior with a convective envelope are subject to advection (i.e. being moved around) by the differential rotation Δ*Ω* between pole and equator that takes place in the envelope. Take the Sun as a representative example, where the differential rotation rate Δ*Ω* is ∼10^−6^ *s*^−1^, and the density *ρ* at the base of the convection zone is 0.2 *g* cm^−3^. Equipartition of magnetic energy density *B*^2^/8*π* with the energy density $\frac{1}{2}\rho {\left(r\Delta \Omega \right)}^{2}$ in this differential rotation corresponds to a field strength of about 10^5^ G. Above this strength, the field would be able to affect differential rotation in the convection zone. Equipartition with the typical energy density in convective flows at the base of the solar convection zone on the other hand would be somewhat less, a few kG.

The typical Ap star field (several kG) would therefore significantly interfere with convection throughout a convective envelope. Even in such a strong field, however, some form of reduced energy transport is likely to exist, because the convective flow and the magnetic field can disengage from each other by the convective expulsion process discussed above. The field gets concentrated in narrow bundles in which flows are suppressed. In the gaps between the bundles the fluid is in a nearly field-free convective state.^10^

An example of how this might work is seen in sunspots, which have surface field strengths well above equipartition with the surrounding convective flows. Below the visible surface of a sunspot a ‘splitting’ process is present in the field configuration, extending to just the visible surface. It produces gaps through which convective flows can transport heat. This explains both the inhomogeneities observed in sunspots, and the relatively large heat flux in sunspots [179,180]. It has convincingly been seen in operation in realistic radiative MHD simulations of sunspots [181–183].

Stars with strong, stable fields and with such sunspot-like phenomenology are not known. The process of accumulation of magnetic flux from a protostellar disc might be different in stars with a final mass like the Sun compared with more massive stars, as the star’s magnetic field could affect the accretion process. However, it seems more likely that survival of a fossil field somehow is not compatible with a convective envelope. If this turns out to be the case, it also raises the possibility that the convective core of an Ap star may affect the evolution of its fossil field. This is discussed further in §3.8.

Convective motions in the envelope would impose random displacements of the field lines extending through the interior. As convective flows have length scales smaller than the stellar radius, the displacements of field lines by these motions are incoherent between their points of entry and exit from the interior. This ‘tangles’ the field lines in the interior: neighbouring field lines get wrapped around each other. This raises the issue of *reconnection*: if in the course of tangling neighbouring field lines can exchange paths by reconnection, the cumulative effect of many such events on small scales would act like an effective diffusion process, allowing field lines to drift at a rate much higher than resulting from microscopic resistivity.

#### 6.2.1. Reconnection

The consequences of this wrapping process have been studied extensively in the context of the solar corona by Parker ([179,184,185] and references therein), who found that it leads to rapid formation of current sheets (on the length and time scale of the displacements), through which reconnection takes place. Numerical simulations of this process have been done for the case of coronal heating of the Sun driven by convective displacements of the footpoints of the coronal field in the photosphere [186]. Using simple MHD simulations, Braithwaite [187] finds this phenomenon not only in a low-*β* plasma (as in the corona) but also in order-unity- (e.g. the ISM) and high-*β* (e.g. stellar interiors) plasmas. Particularly relevant for the present case of reconnection by small-scale flows in a strong background field is the extensive study by Zhdankin *et al.* [188].

The tangling process transports a certain amount of energy from the convection zone into the interior, but more importantly the continued reconnection of field lines in the interior driven by flows in the envelope effectively also acts as an enhanced diffusion of those field lines that are affected by the tangling process.

If the effect is large enough, the field could already disappear from the star when accretion of magnetic flux ends, toward the end of the star’s formation. There is some observational evidence relevant for this, as large-scale fields have been observed also in Herbig Ae-Be stars (cf. §2.1.4 above). Though only a few have been found so far, they occupy the same range of surface temperatures as the main-sequence magnetic Ap stars. The onset of efficient convection around F0 coincides with the disappearance of the Ap phenomenon (e.g. [13]). In the above interpretation it would also mark the onset of enhanced magnetic diffusion. The observations would then imply that the decay of the Ap-type field from a star with a convective envelope is in fact effective on a timescale notably less than the pre-main-sequence life of an F0 star, i.e. less than about 10^7^ yr, or about a factor 100 shorter than the decay time expected from purely Ohmic diffusion.

### 6.3. Convective cores

The above line of thought about enhanced diffusion by ‘tangling’ is also relevant to convection in the cores of the magnetic early-type stars, especially the more massive ones. Going from essentially fully radiative at around 1.5 *M*_⊙_, the convective core extends to around *r*/*R*≈0.16 in late B stars, around 5 *M*_⊙_, and up to around *r*/*R*≈0.3 at even higher masses. In this case, some of the field lines emerging at the surface could pass through the core. In the simple dipolar configuration of figures 9, 11 and 12, these field lines would populate the magnetic poles. The azimuthal field torus that stabilizes the configuration as a whole (§3.3) needs to be located in the stably stratified radiative zone outside the core. Shuffling of field lines by convection could keep the polar field region in a somewhat time-dependent state. This tangling by convective motion may have a similar effect to the tangling of field lines in the solar corona, where it keeps the field above the convective zone close to a potential field. Unlike the case of a convective envelope, however, the stabilizing part of the field is not connected to the convective region; it is unaffected by reconnection processes taking place on the polar field lines. We hypothesize that this explains how Ap star magnetic fields can coexist with a convective core.

Convection in the core would have the secondary effect of exciting some level of internal gravity waves in the surrounding radiative envelope (e.g. [164]). Waves can also increase the rate of magnetic diffusion, but, being periodic, their effect (at the same velocity amplitude) is not comparable with the reconnection processes resulting from the wholesale tangling of field lines discussed above. The magnetic diffusion timescale by Ohmic diffusion alone is of the order of 10^10^ yr in an Ap star, their main-sequence lifetime of the order of 10^8^ yr. A possible wave-induced increase of the diffusion rate by a factor 10–100 would still be compatible with the fields seen in Ap stars.

Another possibly important difference between convective core and convective envelope is the direction of gravity at the boundary between radiative and convective zones. In solar-type stars, cold, fast downflows should penetrate some distance into the radiative zone, but in contrast rising bubbles in convective cores are not expected to overshoot significantly. In addition to this, magnetic fields have an inherent buoyancy as they provide pressure without contributing to density, and have a tendency to rise, either on some thermal timescale (which in most contexts is very long) or on a dynamical timescale if conditions for buoyancy instability [142,143] are met. Both of these effects would tend to make it easier to expel a large-scale field from a convective core, and keep it from re-entering, than to prevent the field in a radiative core from interacting with a convective envelope. It may be that fossil fields do not enter the convective core. The structure and stability of the non-axisymmetric equilibria in particular (figure 12*b*) would be little affected by expulsion from the core.

Convective cores are expected to host a dynamo; this has been seen in the simulations of Browning *et al.* [189] and Brun *et al.* [190]. Interestingly, a fossil field in the radiative zone might have an important effect on the nature of the core dynamo. Featherstone *et al.* [191] performed simulations of a core dynamo in the case where the surrounding radiative zone contains a fossil field. Without a fossil field, an equipartition field is generated in the core; the addition of a fossil field (significantly weaker than this equipartition strength) switches the dynamo to a different regime in which the field generated is much stronger, which not surprisingly changes the properties of the convection. This would be analogous to the situation in accretion discs, where the presence or absence of even a weak net flux through the disc appears to have a fundamental effect on the nature of the dynamo. This result could be relevant for any compact stellar remnant born out of the core, as it would enable neutron stars and perhaps also white dwarfs to inherit in some way the magnetic properties of their progenitors.

### 6.4. Fully convective stars

**Figure 17.**
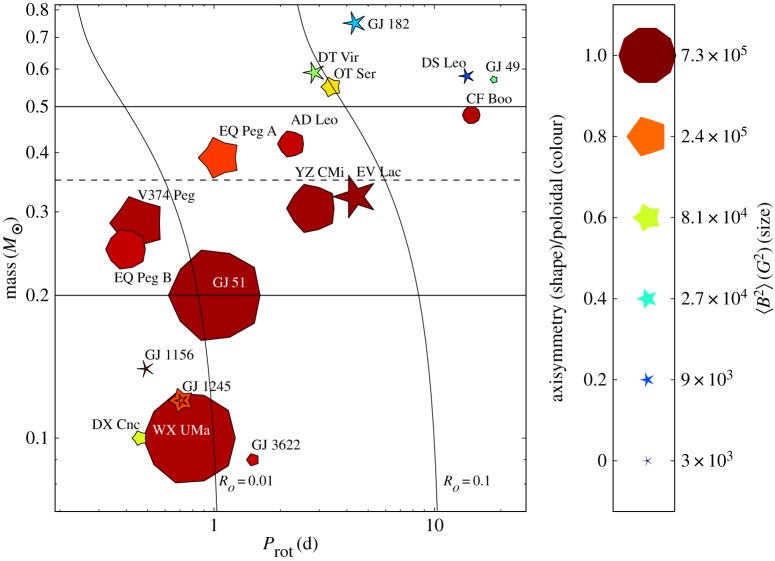
Properties of the surface distribution of the magnetic field (derived from Zeeman–Doppler Imaging) of the M dwarfs observed with the spectropolarimeters ESPaDOnS (Canada–France–Hawaii Telescope) and NARVAL (Télescope Bernard Lyot) as a function of rotation period and mass. Larger symbols indicate stronger fields, symbol shapes depict the degree of axisymmetry of the reconstructed magnetic field (from decagons for purely axisymmetric to sharp stars for purely non-axisymmetric), and colours the field configuration (from blue for purely toroidal to red for purely poloidal). Solid curves represent contours of constant Rossby number *Ro*=0.1 (saturation threshold) and 0.01. The theoretical full-convection limit (0.35 *M*_⊙_) is plotted as a horizontal dashed line, and the approximate limits of the three stellar groups discussed in the text are represented as horizontal solid lines. Compiled from the studies by Morin *et al.* [192,195,196], Donati *et al.* [197] and Phan-Bao *et al.* [198].

In the light of interesting recent observations of pre-main-sequence stars and low-mass stars, in this section we deviate from the main focus of this article—non-convective zones—to discuss briefly magnetic fields in fully convective stars. In contrast to solar-type stars, both main-sequence stars below about 0.4 *M*_⊙_ and T Tauri stars often display dipole fields of the order of 1 kG (e.g. [192–194] and references therein). Some recent results are summarized in figure 17. Gregory *et al.* [199] find that fully convective pre-main-sequence stars tend to have strong dipolar fields roughly aligned with the rotation axis. Those which have a small radiative core tend to have both strong dipolar and octupolar components, and those with larger radiative cores have more complex fields and only a weak dipole component. Intuitively, this is perhaps not surprising as it is difficult in a thin convective envelope to get different parts of the envelope to ‘communicate’ with each other and form a global magnetic field. The length scale of sunspot systems is comparable with the depth of the convective layer; extrapolation to a fully convective star might explain the large scale of their field.

Browning [200] performed simulations of a fully convective star, finding that a dipole-like field can indeed be generated. It is thought that the rotation is a key ingredient in producing a coherent large-scale field from smaller-scale motion, as in standard mean field dynamo models.

One can make an analogy here with planetary magnetic fields. The Earth, Jupiter and Saturn all contain convective, conducting fluids: the Earth (the outer core) between about 0.19 and 0.44*R*_E_, Jupiter from 0 or 0.1 out to 0.78*R*_J_ and Saturn between about 0.15 and 0.5*R*_S_. Important for the nature of the dynamo is the ratio between these inner and outer radii, and in that sense these planets are analogous to stars with small radiative cores. Looking at the results of Gregory *et al.* [199], we should expect Jupiter to have a predominantly dipolar field aligned with the rotation axis, which it does. We expect Earth and Saturn to have probably also a significant octupole component, but because this drops off faster with radius in the overlying non-conducting fluid than the dipole component, we would still expect the field to be predominantly dipolar at the surface and approximately aligned with the rotation axis, which is also consistent with observations.

By analogy though with the Earth’s dynamo, where the polarity of the field changes, we may speculate that the same happens in low-mass stars, in which case even the strong dipole fields observed would not be steady like those in radiative stars.

### 6.5. Subsurface convection in early-type stars

**Figure 18.**
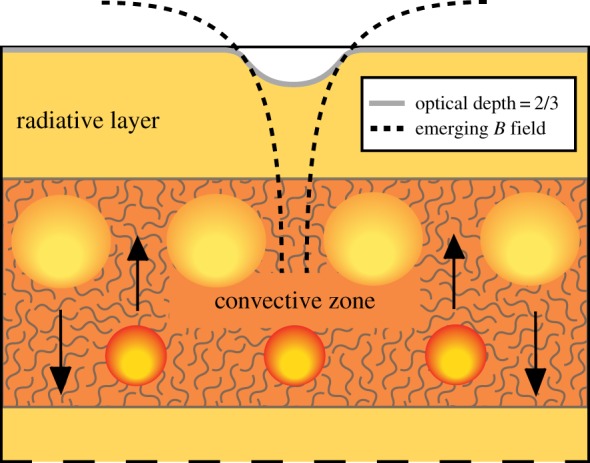
Schematic of the magnetic field generated by dynamo action in a subsurface convection zone. Note that magnetic features should appear as bright spots on the surface, rather than as dark spots as in stars with convective envelopes. From Cantiello & Braithwaite [202].

In early-type stars, while it seems very unlikely that a magnetic field generated by a dynamo in the convective core could rise all the way to the surface on a sensible timescale, these stars also have convective layers close to the surface which could in principle generate fields, which then rise to the surface. The convection is driven by bumps in the opacity at certain temperatures, caused by the ionization of iron, helium and hydrogen. Massive stars (above about 8*M*_⊙_) have two or three such layers, the deepest and energetically most interesting of which is driven by iron ionization [201]. If this layer hosts a dynamo, there is no difficulty for the resulting magnetic field to reach the surface very quickly via buoyancy, as the thermal timescale is so short at the very low density in the overlying radiative layer [202] (figure 18).

At the surface of the star, magnetic pressure supports magnetic features against the surrounding gas pressure, meaning that at a given height, the gas pressure inside the magnetic features is lower than in the surroundings. As the photosphere is approximately located where the column density of gas above it has a certain value, the photosphere in magnetic features is lower than in the surroundings. In a radiative star, this means that magnetic spots appear bright. This contrasts with convective stars where the magnetic field has the additional effect of suppressing convection and therefore heat transfer, and magnetic spots are dark.

**Figure 19.**
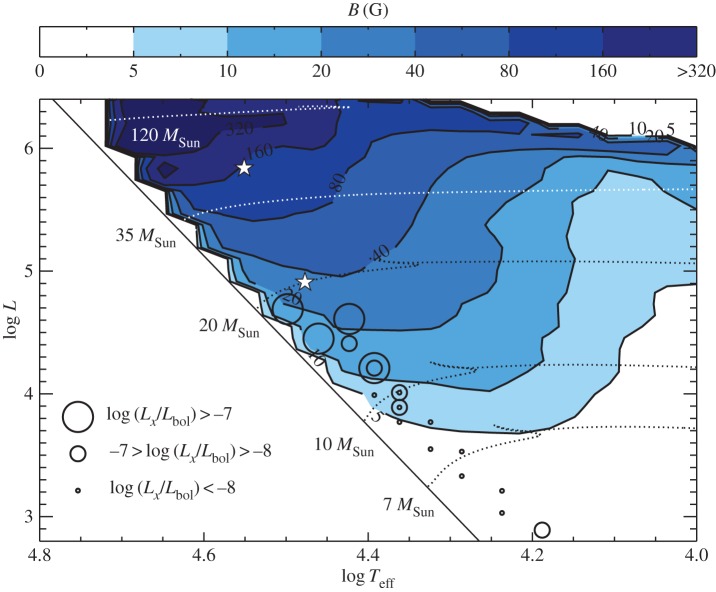
The field strength predicted at the surface of massive stars. The prediction assumes an equipartition dynamo in the convective layer and a *B*∝*ρ*^2/3^ dependence in the overlying radiative layer. Also shown are the X-ray luminosities measured in a number of stars: there does seem to be some connection between subsurface convection and X-ray emission. The dotted lines are evolutionary tracks of stars of various initial masses. From Cantiello & Braithwaite [202].

For solar metallicity, field strengths of approximately 5 to 300 G are predicted—as depicted in figure 19. The field strength depends on the mass and age of the star: higher fields in more massive stars and towards the end of the main sequence. These fields are expected to dissipate energy above the stellar surface and could give rise to, or at least play some role in, various observational effects such as line profile variability, discrete absorption components, wind clumping, solar-like oscillations, red noise, photometric variability and X-ray emission (see, e.g. [203] for a review of these phenomena). Indeed, if the X-ray luminosities of various main-sequence stars are plotted on the HR diagram (figure 19) a connection with subsurface convection does seem apparent. Of course, we cannot be certain that it is a magnetic field which is mediating transfer of energy and variability from the convective layer to the surface; one could also imagine that internal gravity waves are involved. Simulations of the generation and propagation of such gravity waves were presented by Cantiello *et al.* [204].

#### 6.5.1. Intermediate-mass stars

Stars below about 8*M*_⊙_ have no such iron-ionization-driven convective layer, but do have similar layers caused by helium and hydrogen ionization. In intermediate-mass stars such as Vega and Sirius (spectral types A0 and A1, respectively), a dynamo-generated field could float to the surface from a helium-ionization-driven convective layer beneath the surface [205]. Field strengths of a few gauss are predicted. With the observations done so far though, it might be difficult to distinguish between this and the failed fossil hypothesis [115] described above in §3.7. The main difference would be the length scales: the convective layer is very thin and it would be difficult to generate magnetic features large enough to be detectable as a disc-averaged line-of-sight component (cf. [206]). In comparison with more massive stars, late-B and early-A stars are very quiet in X-rays, and so far only upper limits on X-ray emission have been established. For instance, Pease *et al.* [207] obtained an upper limit of 3×10^25^ erg s^−1^ in X-rays from Vega. Pre-main-sequence stars in this mass range are known to emit X-rays, but Drake *et al.* [208] obtain a limit of 1.3×10^27^ erg s^−1^ from the 8 Myr old A0 star HR 4796A, demonstrating that this X-ray activity shuts down roughly when the star reaches the ZAMS. This is consistent with weak dynamo activity; Drake *et al.* [208] predict an X-ray luminosity of very approximately 10^25^ erg s^−1^ from magnetic activity in Vega, and lower luminosites in slower rotators. Cooler than around type A5 (*T*_eff_∼10 000 *K*), X-rays become detectable again, presumably owing to convection at the surface; this convection is also detected more directly as microturbulence [209].

#### 6.5.2. Interaction with fossil fields

The various observational phenomena in massive stars, listed above, seem to be ubiquitous. Notably, they are present also in stars in which strong large-scale fields have been detected. This means that if these phenomena are caused by subsurface convection, this convection is not disturbed significantly by the fossil field. However, a fossil field of the order of 1 kG is in equipartition with the predicted convective kinetic energy and should at least have some effect on it (e.g. [202]). The magnetic field may simply force the entropy gradient to become steeper until convection resumes. This is often assumed in parametrizations of the effect of magnetic fields in stellar evolution calculations (e.g. [210]). As discussed above (§6.2), however, interaction of convection with fields at strengths of the order of equipartition is very inhomogeneous, and even small gaps between strands of strong field can allow a nearly unimpeded convective heat flux. The effects of such inhomogeneous fields on stellar structure are much smaller than in conventional parametrizations based on average field strengths [211,212].

In the case of the early-type stars (§6.5), subsurface convection only transports a modest fraction of the total energy flux and one could imagine it is easier to suppress. There is recent observational evidence that a very strong magnetic field can indeed suppress the subsurface convection: Sundqvist *et al.* [213] measure macroturbulent velocities in a sample of magnetic OB stars, finding that one star in the sample (NGC 1624-2), which has a field of around 20 kG, lacks significant macroturbulence, while the rest, which have fields up to around 3 kG, have vigorous macroturbulence of over 20 km s^−1^. The thermal energy density in the convective layer corresponds to an equipartition field of around 15 kG, so this appears to confirm that convection can be suppressed only by a field comparable in energy density to the thermal—rather than convective kinetic—energy density, even when convection is weak. Obviously, it would be useful to improve the observational statistics, and to look at stars with fields between 3 and 20 kG. Finally, while it seems likely that macroturbulence (the part thereof which is not the result of stellar rotation) is essentially gravity waves produced by subsurface convection, the origin of the various other observational phenomena in massive stars is less certain; it would therefore be a very useful to determine whether strongly magnetic stars lacking macroturbulence display these other phenomena.

In intermediate-mass stars, there is also some evidence that fossil fields suppress convection. Although late-A stars normally display microturbulence, it seems to be lacking in the magnetic subset of the population (Shukyak 2013, personal communication). However, this is perhaps not directly comparable to massive stars because the surface convection is very weak, and all fossil fields are at least an order of magnitude stronger than equipartition with the convective kinetic energy.

## 7. Neutron stars

**Figure 20.**
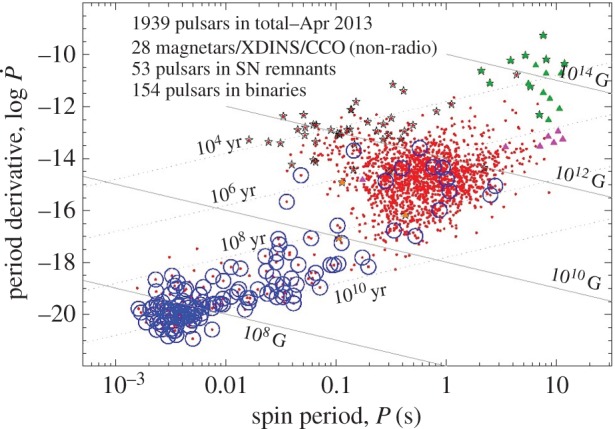
The well-known $P-\dot{P}$ diagram on which two readily measurable quantities are plotted, period derivative against period, together with inferred magnetic dipole field strengths and spindown ages (diagonal lines). The main clump is the radio pulsars (red dots), of which around 2000 are known. At the top right are the magnetars (green triangles), and on the lower left we have old neutron stars with very weak dipole fields which have been spun up by accretion. Blue circles represent binary systems and stars supernova remnants. The so-called magnificent seven are the pink triangles on the right. Figure provided by T. M. Tauris.

Field strengths of neutron stars observed as pulsars and magnetars span the range of 10^10^–10^15^ G (see figure 20). (In addition, there are the ‘recycled millisecond pulsars’, with field strengths around 10^8^ G. Their fields are believed not to be representative of their formation, instead representing a process related to the recycling. See Harding [214], for a review of different classes of neutron star.) The width of this range—several decades—is similar to that seen in mWD and Ap stars. The presence of a solid crust makes a difference compared with the other classes, however, as it can anchor fields that otherwise would be unstable. Before this anchoring takes place, the magnetic field of the proto-neutron star is subject to the same instabilities and decay processes as in Ap stars. This is discussed below in §7.2.

### 7.1. Mechanisms of field generation

Three mechanisms have been proposed. The field could be inherited from the pre-collapse core of the progenitor star (the ‘fossil’ theory^11^), it could be generated by a convective dynamo during (or shortly after) core collapse, or it could be generated by differential rotation alone, via a magnetorotational mechanism like that operating in accretion discs. This mechanism looks especially promising for field generation during or shortly after collapse of a rotating core (cf. [215]).

#### 7.1.1. Inheritance

In this scenario, the field is simply a compression of what was present in the progenitor, which may be either an evolved high-mass star or an accreting white dwarf. We discuss first the pre-collapse evolution of massive stars.

**Figure 21.**
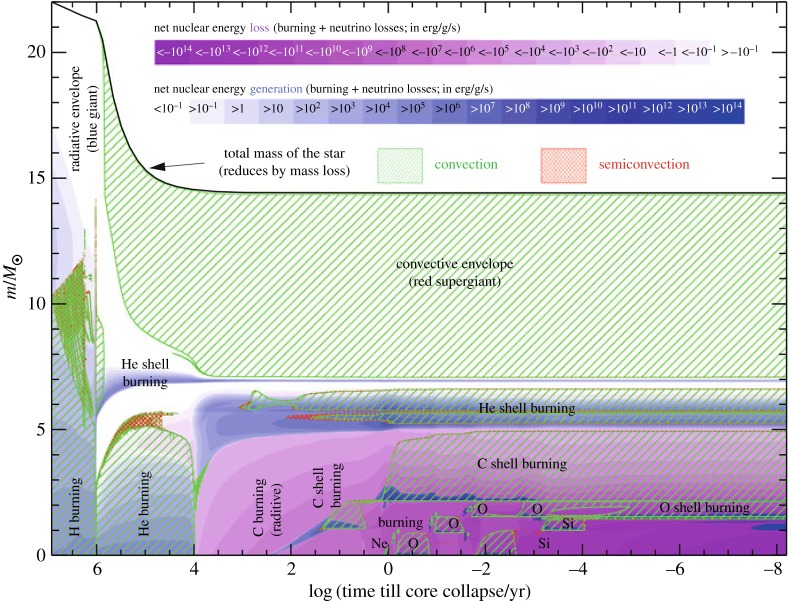
A Kippenhahn diagram of a star with an initial mass 22*M*_⊙_. Along the horizontal axis is log time before the supernova, so the main sequence takes up only a short space on the left. Note the appearance and disappearance of convective layers. Figure provided by A. Heger.

During the main sequence, the core of the star is convective, and afterwards various convective zones appear and disappear at different locations as burning moves in steps to heavier elements, as is illustrated in figure 21 [216] (Heger 2013, personal communication). These convective zones may become relevant to the magnetic field of the star, especially in the core, out of which the neutron star will eventually form. During the main sequence, we expect an active dynamo in the core, perhaps similar to those in low-mass stars. For instance, Browning *et al.* [189], Brun *et al.* [190] and Featherstone *et al.* [191] performed simulations of an A-star convective core, finding a sustained dynamo. After the main sequence, the star moves onto the red-giant branch. Magnetic fields have been observed in several red giants, such as EK Boo, an M5 giant, where a field of *B*∼1–8 G was detected [217]. Another example is Arcturus, a K1.5 III giant, where a subgauss field has been found [218]. The nature of these fields is poorly constrained at present but they are presumably generated by a dynamo in the convective envelope, and may or may not be directly relevant for the neutron star’s magnetic field.

Under flux freezing (see also §3.1), the field in a pre-collapse core of radius *R*_0_∼3×10^8^ cm collapsing to a neutron star of radius *R*=10^6^, will be amplified by factor 10^5^. To explain magnetar strength fields of 10^15^ G (more realistically, internal fields of 10^16^, see §7.3), requires an initial field *B*_0_ of 10^10^ G. This is more than a factor 10 larger than the largest field strengths seen in magnetic white dwarfs (which have about the same size and mass as such cores). The question what could lead to such a strong field in the progenitor core is still open.

In the accretion-induced collapse (AIC) scenario, a white dwarf passes over the Chandrasekhar mass limit and collapses into a neutron star, the composition of the star being such that there is insufficient nuclear energy to produce a supernova. Accreting white dwarfs have a variety of magnetic properties, with large-scale fields observed with strengths up to at least 10^8^ G. This does not seem to be quite sufficient to explain fields in magnetars.

#### 7.1.2. Convection

Thompson & Duncan [219] proposed that magnetic fields in neutron stars are generated in the young neutron star (NS) by a dynamo deriving its energy from the convection with the help of the differential rotation, estimating an upper limit to the field strength of 3×10^15^ G, a little above the highest dipole component measured in SGRs and AXPs. A challenge for the theory is how to transfer this energy from the length scale of convection (around 1 km) to the dipole component on 10 times that scale. If magnetic helicity is conserved (see §4), then most of the energy should be lost once convection dies away; in fact, even if the magnetic field in every convective element is twisted in the same direction in some sense (corresponding to maximum helicity) then one still expects to lose 90% of the energy (a reduction in field strength of a factor of 3) if the dominant length scale is to rise by a factor of ten. If symmetry breaking does not work and convective elements are twisted in random directions then one loses a further factor of $\sqrt{N}$ of the energy. It is a general challenge for dynamo theory to produce large-scale structures, but the problem does not seem insurmountable: rotating fully convective stars are observed to have dipolar fields [192] and the same has been reproduced in simulations (e.g. [200]); see also §6.4. It may be that magnetars were born very quickly rotating; energetically, one is in a much more favourable position if the star is rotating with a period of 3 ms or 1 ms at birth. However, one expects such a fast-rotating, highly magnetized NS to spin down within a day or so, or even faster if it has a strong wind, injecting 2×10^51^ to 2×10^52^ erg into the supernova and creating a ‘hypernova’. There is evidence from supernovae remnants that this is not the case: Vink & Kuiper [220] compared energies of SN remnants around magnetars and other neutron stars and found no significant difference, concluding that the spin period of magnetars at birth must be at least 5 ms.

#### 7.1.3. Differential rotation

Energy in the differential rotation could also be tapped by the magnetorotational instability (MRI; [84,141,221]). Its importance in the context of neutron star magnetic fields comes from the exponential growth of MRI on a differential-rotation timescale. It may be most relevant in the proto-neutron star, after the convection has finished but when the star is still differentially rotating.

In main-sequence stars, the dynamo mechanism proposed by Spruit ([148]; see also [150]) should dominate over the MRI as it is inhibited less by the strong entropy stratification and works better than the MRI when differential rotation is weak. In proto-neutron stars, which probably have quite strong differential rotation, it could also convert some of the energy in the differential rotation into magnetic form, but it works more slowly because it involves an initial amplification stage where the field strength increases only linearly with time. The time available in the collapse and immediate post-collapse phase is likely to be insufficient for this process. In proto-neutron stars, field generation from the magnetorotational instability is a more likely process.

In any case, the fact that older neutron stars are known to have magnetic fields, despite having used up their natal energy reservoir of differential rotation, brings us to the next section.

#### 7.1.4. Magnetic helicity

In any case, important for generation of a magnetar field will be the magnetic helicity present, as when the driving processes switch off, the star will try to relax into a minimum energy state for its level of helicity. The process that determines the helicity produced by the combination of a field amplification process, as well as the buoyant instabilities that bring the field to the surface of the star, are unclear. This is just as in the case of the Ap and mWD stars.

Helicity need not be conserved if the field is brought to the surface as we only expect conservation in a highly conducting medium, so it may still be possible to build or destroy helicity during the star’s early evolution. To build helicity, the symmetry (between positive and negative helicity, or in other words between right- and left-handed twist), has to be broken.

In terms of explaining the apparent diversity in neutron star properties, beyond the two degrees of freedom which place the star on the $P-\dot{P}$ diagram, it helps to think in terms of the range in available magnetic field configurations (§7.3).

### 7.2. Field evolution before crust formation

When neutron stars form they are differentially rotating and convective in most of the volume. As the star cools by neutrino emission the stratification becomes stable. Some time later—estimates vary from 30 s to 1 day after formation—a solid crust forms (e.g. [222] and references therein). At some stage (either before or after crust formation), any field-generating dynamo present will die away, after which time the field will relax towards an MHD equilibrium; this happens on a dynamical timescale, the Alfvén timescale *τ*_A_. This timescale is short, ranging from 3 h down to 0.1 s for field strengths ranging from 10^10^ to 10^15^ G, respectively, so in a non-rotating star one would expect that in most cases an MHD equilibrium must be established before the crust forms. Rotation, however, should slow down the formation of equilibrium to a timescale given by $\tau_{\mathrm{evol}}∼\tau_{A}^{2}\Omega$, where *Ω* is the angular rotation velocity of the star (see [115], and §3.7 for a discussion of this effect in A stars). Assuming an initial rotation period of 30 ms, an equilibrium would then take 600 years to form for a field of 10^10^ G or 2 s for a field of 10^15^ G. So it may be that only the magnetars really find an MHD equilibrium before the crust forms. Making an estimate of the strength of a crust (shear modulus and breaking strain, see e.g. [223,224]), it seems that a non-equilibrium magnetar-strength field would not be stopped from evolving anyway, in contrast with radio-pulsar-strength fields which could be held in position ‘against their will’, so to speak. Of course it might not be a coincidence that most neutron stars have fields at about the crust-breaking threshold; the magnetic field decays until it reaches this threshold and is prevented from decaying further. In this picture the magnetars would, for some reason, be born with a greater magnetic helicity than the other neutron stars; however, the so-called central compact objects (CCOs) with fields of 10^10^ G would indicate intrinsically less efficient field generation. In any case, the location of the crust-breaking threshold is subject to large uncertainty, because of our poor understanding of the properties of the crust and perhaps, more importantly of the geometry of the magnetic field and crustal fracturing.

### 7.3. The energy budget of magnetars

There is a consensus that magnetars are powered by the decay of their magnetic field, whereby it is necessary for energy to be dissipated in or above the crust rather than the interior, to avoid losing the energy to neutrinos (e.g. [225]). A magnetar with an RMS strength 10^15^ G in its interior contains around 3×10^47^ erg in magnetic energy, enough to maintain a mean luminosity of 3×10^35^ erg s^−1^ for a lifetime of 3×10^4^ yr (e.g. [226], for a review of the observations). However, we have now seen giant flares in three objects, the most energetic of which is thought to have released (very approximately) 3×10^46^ erg in just a few seconds; if it is not to be a coincidence to have observed this many giant flares then the energy source needs to be larger. In addition, most SGRs and AXPs have somewhat weaker measured dipole fields than 10^15^ G, some are below 10^14^ G. It looks as if the field strength in the interior of the star needs to be greater than we infer from the spindown rate, which gives us just the dipole component at the surface.

Fortunately, this seems very possible. It may be that the magnetars have a strong toroidal field in relation to the poloidal component which emerges through the surface; Braithwaite [90] and Akgün *et al.* [91] found that for a given poloidal field strength a much stronger toroidal field is permitted, as the stratification hinders radial motion and therefore also the instability of a toroidal field. Also the magnetar fields could be more complex than a simple dipole; Braithwaite [93] found a range of non-axisymmetric equilibrium where a measurement of the dipole component gives an underestimate of the actual field strength. Alternatively, the field could be largely buried in the stellar interior with only a small fraction of the total flux actually emerging through the surface; whether this is possible depends both on where the field is originally generated and on diffusive processes which bring the field towards the surface over long timescales [93,108]. In any case, there are various degrees of freedom available (see §3).

## 8. Summary and open questions

The subject of magnetic fields in the interior of stars inevitably relies to a large extent on theoretical developments. The increasing quality and quantity of observational constraints, however, is providing more clues and constraints on theory than ever before. In parallel, the increasing realism of numerical MHD simulations makes them an effective and indispensable means of testing theoretical speculation. An example is the subject of ‘fossil fields’ that have become the preferred interpretation of the steady magnetic fields seen on Ap, Bp and O stars. Here, numerical MHD has not only convincingly reproduced the range of observed surface distributions of these stars, but also provided physical understanding of their stability and internal structure. Axisymmetric purely toroidal or purely poloidal fields are unstable, and so they must exist together, in a certain range of strength ratios; the two components can either be comparable with each other, or the toroidal field can be stronger. The average interior field of a neutron star could be much higher than the surface dipole component inferred from spindown, which would explain how magnetars appear to have a more generous energy budget than one would estimate from their dipole components alone.

A key to understanding the nature of such stable equilibria is magnetic helicity (§§3.6.1, 4.4 and 7.1.4). To the extent that it is conserved during relaxation of a field configuration, it guarantees the existence of stable equilibria of finite strength: a vanishing field has vanishing helicity. An open issue, however, concerns *helicity generation*: the question of which mechanism(s) has/have given the field the magnetic helicity that is essential for its long-term survival. Perhaps, stochastical coincidences during the dynamical phases of star formation and evolution (§4.3) may be all that is needed.

The recent finding that the fields in Ap stars appear to have a minimum of about 200 G (§2.1) may be a clue, still to be deciphered, for the formation mechanism of the fields. The much lower fields of the order of a gauss discovered in the two brightest A stars, on the other hand, may just be the result of an initially stronger field that is presently still in the process of decaying (§2.2.1).

Stars in the A-B-O range have convective cores, which are likely to interact to some extent with the stable fossil field in (the remainder of) the star. This is still a very open question. Some inconclusive speculations on the physics that may be involved in such interaction were given in §6.3. The question may have an observational connection, however. The distribution of field strengths of ABO stars across the HRD has recently been shown to indicate decay on a somewhat shorter timescale than can be attributed to finite (‘Ohmic’) resistivity; perhaps, an indication of enhanced diffusion related to interaction with the convective core, or with a convective envelope developing as the star evolves off the main sequence (§§3.8 and 6.3). In stars going through a fully convective phase, any fossil field (such as that inherited from a molecular cloud) should be lost by magnetic buoyancy. Common to ABO stars, white dwarfs and neutron stars are the large range in field strengths in the population, and the ratio of magnetic to gravitational energy, which ranges from about 10^−16^ to 10^−6^ in all three classes of star. The size of this range is a puzzle for any theory of the origin of these magnetic fields (§§2.1 and 4.1).

Solar-type stars do not show magnetic fields remotely resembling the stable Ap configurations and strengths. This has led to the speculation that the radiative interiors of these stars might still have a fossil field, but shielded from the surface by the convective envelope. Such a field can then be invoked to explain the near-uniform rotation of the Sun’s radiative interior. A clear distinction has to be made here between *shielding* by the convective envelope and *decoupling* from it (§6.1). Convective processes are known that could shield an internal field from becoming observable at the surface, but in the presence of magnetic diffusion it is impossible to avoid mechanical coupling across the boundary between interior and envelope. It causes the internal field to evolve on timescales governed by interaction with the differential rotation of the envelope; the result will probably look more like the differential rotation-driven dynamo process discussed in §5.4.

Further clues on magnetic fields in the interiors of stars come from asteroseismic results on the internal rotation of giants and sub-giants (§5.5.1). The coupling between core and envelope deduced from these results is far stronger than can be explained with existing hydrodynamic coupling processes, such as shear instabilities. In stably stratified zones of stars, time-dependent self-sustained magnetic fields powered only by differential rotation and magnetic instabilities (i.e. ones governed by the Maxwell stress) are likely to operate, except in very slowly rotating stars. The favoured scenario for such a dynamo process (§5.4) fares much better in matching the asteroseismic observations, but still misses the target by a significant factor. Magnetic fields, while probably involved in stably stratified zones of stars, may have modes of behaviour not yet recognized.

Future progress on the questions raised by the observations is likely to benefit increasingly from numerical simulations. The main obstacle is the fact that in almost all cases simulations for the actual physical conditions in stellar interiors will remain out of reach in the foreseeable future, irrespective of expected increases in computing power. Experience shows that extrapolation over the missing orders of magnitude in physical parameter space cannot be done simply from the simulations themselves. Extrapolation needs physical understanding formalized in models that cover the asymptotic conditions encountered in stars. The validity of such models can often be tested well with targeted simulations of reduced scope (i.e. not with ‘three-dimensional stars’). The formulation of the models themselves requires more classical style theoretical effort; however, compare the discussion on p. 11 in [227].

## Footnotes

1 The term ‘chemical elements’ is used anomalously in astrophysics (including this review) to mean atomic species.

2 The common usage of the term ‘topology’ in this context is sloppy. Its meaning is distribution on the star’s surface. Topology is by definition a global property of the entire field configuration; nothing can be inferred about it from observations of the stellar surface alone.

3 The title of the paper incorrectly says ‘decrease’.

4 This is not possible in a hypothetical convectively neutral star where entropy is constant. In this case, the field must be of the much more restricted class for which the Lorentz force has a potential. See also §3.6.

5 In the literature, one often finds that the factor of 2 inside the brackets is missing.

6 Note that *β*∼λ^2^(*h*/*r*)*M*_*_/*M*_disc_, where $\beta =8\pi P/\bar{B^{2}}$; in a disc, therefore, it is possible to have both *β*>1 and λ<1 at the same time, in contrast with stars, where *β*∼λ^2^.

7 The two uses of the term ‘dynamo’ in the literature may lead to confusion here. The process is not a global dynamo as envisaged in ‘mean field’ models.

8 The standard recipes used in stellar evolution calculations ‘with rotation’ in fact fail this constraint rather spectacularly, when applied to the Sun.

9 The model does not include the radial advection and diffusion of the poloidal component of the magnetic field (*B*_p_). It is instead replaced by an assumed fixed value *B*_0_. The model includes an equation for diffusion of the azimuthal field component (*B*_*ϕ*_), but leaves out its radial advection by the circulation.

10 This separation is reminiscent of the formation of flux bundles in a superconductor in an imposed magnetic field, cf. http://hyperphysics.phy-astr.gsu.edu/hbase/solids/scbc.html.

11 There is some inconsistency in the literature concerning the meaning of ‘fossil field’. In the Ap-star context, it is normally taken to mean a field left over from an earlier epoch, for instance the pre-main-sequence or the parent ISM cloud, rather than being the result of some contemporary dynamo process. In the neutron star context, it means not just this but also that the earlier epoch is not the proto-neutron star phase but the pre-collapse progenitor.
